# TALEN/CRISPR-Mediated eGFP Knock-In Add-On at the *OCT4* Locus Does Not Impact Differentiation of Human Embryonic Stem Cells towards Endoderm

**DOI:** 10.1371/journal.pone.0114275

**Published:** 2014-12-04

**Authors:** Nicole A. J. Krentz, Cuilan Nian, Francis C. Lynn

**Affiliations:** 1 Diabetes Research Program, Child and Family Research Institute, Vancouver, British Columbia, Canada; 2 Department of Surgery and Department of Cellular and Physiological Sciences, University of British Columbia, Vancouver, British Columbia, Canada; University of Tampere, Finland

## Abstract

Human embryonic stem cells (hESCs) have great promise as a source of unlimited transplantable cells for regenerative medicine. However, current progress on producing the desired cell type for disease treatment has been limited due to an insufficient understanding of the developmental processes that govern their differentiation, as well as a paucity of tools to systematically study differentiation in the lab. In order to overcome these limitations, cell-type reporter hESC lines will be required. Here we outline two strategies using Transcription Activator Like Effector Nucleases (TALENs) and Clustered Regularly Interspaced Short Palindromic Repeats (CRISPR)-CRISPR-Associated protein (Cas) to create OCT4-eGFP knock-in add-on hESC lines. Thirty-one and forty-seven percent of clones were correctly modified using the TALEN and CRISPR-Cas9 systems, respectively. Further analysis of three correctly targeted clones demonstrated that the insertion of eGFP in-frame with OCT4 neither significantly impacted expression from the wild type allele nor did the fusion protein have a dramatically different biological stability. Importantly, the OCT4-eGFP fusion was easily detected using microscopy, flow cytometry and western blotting. The OCT4 reporter lines remained equally competent at producing CXCR4+ definitive endoderm that expressed a panel of endodermal genes. Moreover, the genomic modification did not impact the formation of NKX6.1+/SOX9+ pancreatic progenitor cells following directed differentiation. In conclusion, these findings demonstrate for the first time that CRISPR-Cas9 can be used to modify *OCT4* and highlight the feasibility of creating cell-type specific reporter hESC lines utilizing genome-editing tools that facilitate homologous recombination.

## Introduction

Embryonic stem cells (ESCs) are pluripotent cells located in the inner cell mass of early embryos that have the capacity for long-term self-renewal and the ability to form all cell types of the embryo proper. Since the generation and successful culture of the first human (h)ESC line [1], there has been great excitement surrounding their potential to treat many diseases, including diabetes [1]–[4]. Unfortunately, progress in making fully functional terminally differentiated cells has been slow. This is likely due to both the insufficient knowledge of the developmental processes that govern tissue formation and the lack of appropriate tools to study development in culture [5], [6]. One potential method to address both of these issues is the generation of reporter hESC lines that facilitate the study of human development in culture and to allow for high throughput, high content screens to uncover factors that drive differentiation.

Previously, creation of reporter hESC lines has primarily been limited to transgenesis using constitutive [7]–[12] or truncated promoters [7], [13], [14]. These strategies are not ideal, as variation in copy number and integration sites may affect expression of reporter genes. More importantly, there is a significant likelihood of transgene silencing upon differentiation, especially to more specialized cell types [15], and a risk of disrupting endogenous gene expression. Another strategy is to replace one allele with the reporter gene; however, this creates haploinsufficiency that can impair differentiation and complicate interpretation. A better approach is to knock-in a reporter gene downstream, but in-frame with the protein of interest, allowing for marker expression driven by the endogenous promoter without altering expression of the targeted gene. This strategy was previously difficult in hESCs due to the low rate of homologous recombination and the requirement for very large homology arms [16]. With the recent advent of three high efficiency genome editing technologies, Zinc Finger Nucleases (ZFNs), Transcription Activator Like Effector Nucleases (TALENs) and Clustered Regularly Interspaced Short Palindromic Repeats (CRISPR)-CRISPR-Associated protein (Cas), genome editing is fast becoming a reality in human ESCs [17]–[19]. These technologies utilize sequence-specific (10–30 bp in length) nucleases to create a double stranded break in the DNA, which dramatically increases the frequency of homologous recombination through homology directed repair. While several landmark papers have described the generation of reporter lines using these technologies [20]–[22], no studies have thoroughly investigated the effects of the genomic modification on stem cell characteristics or directed differentiation potential.

*Oct4/Pou5f1* is a key member of the pluripotency network [23] and while *Oct4^−/−^* embryos develop to the blastocyst stage, they do not contain pluripotent cells within the inner cell mass [24]. In addition to its role in maintaining embryonic stem cell pluripotency, Oct4 is also important for differentiation, as *Oct4* expression is required for the formation of all embryonic lineages *in vitro* and *in vivo* [25]. For instance, in zebrafish the *Oct4* homolog is essential for endoderm formation [26] and maternal-zygotic *Oct4* mutant embryos display delayed gastrulation and absence of endoderm [27].

The dual role of Oct4 in both maintaining pluripotency and establishing endoderm is believed to be driven by its Sox binding partner. Oct4 interacts with Sox2 at “canonical” binding sites to maintain pluripotency, while endoderm specification involves Oct4 and Sox17 binding at “compressed” Sox/Oct motifs [28]. Consistent with this finding, point mutations in the Oct4-interaction interface of Sox17 allow Sox17 to cooperate with Oct4 at canonical sites and drive reprogramming; whereas, mutations to the analogous region of Sox2 allow it to cooperate with Oct4 to drive endoderm formation [29]. Consistent with the role of Oct4 in mouse development, it appears that the level of human *OCT4* expression dictates which lineage stem cells will differentiate towards: reduced expression of *OCT4* promotes the mesoderm lineage while elevated OCT4 promotes adoption of the endoderm lineage [30]. The critical role of OCT4 in endoderm formation suggests that any changes in *OCT4* expression or stability in hESC reporter cell lines may alter their differentiation potential, especially to endodermally-derived tissues.

In the present study both TALEN and CRISPR-Cas approaches were used to generate OCT4-eGFP-2A-Puro reporter lines using the CyT49 hESC line: the CRISPR-Cas9 strategy being slightly more efficient. In order to understand if *OCT4* targeting impacted stem cell identity or the ability of hESCs to form endoderm and endodermally-derived tissues, three of the correctly targeted clones were analyzed further. Knock-in add-on of eGFP to *OCT4* did not affect pluripotency; eGFP fluorescence mirrored expression of OCT4; and genomic modification did not alter expression from the wild type *OCT4* allele. Finally, genomic modification of *OCT4* did not alter the potential of these cells to differentiate either to definitive endoderm or towards downstream pancreatic progenitor cells. Taken together, these results support the use of genome editing technologies to efficiently generate reporter hESC lines.

## Materials and Methods

### Cell culture

Undifferentiated CyT49 hESCs (ViaCyte, Inc. San Diego CA) were maintained on EmbryoMAX Primary Mouse Embryo Fibroblasts (MEF) feeder layers (Millipore) in 10/10 media [DMEM/F12 (Cellgro), 10% XenoFree KnockOut Serum Replacement (Life Technologies), 1x MEM non-essential amino acids (Life Technologies), 1x GlutaMAX (Life Technologies), 1x penicillin/streptomycin (10,000 U/mL) (Life Technologies), 10 nM β-mercaptoethanol (Sigma), supplemented with 10 ng/mL Activin A (R&D) and 10 ng/mL Heregulin-β1 (Peprotech)] [31], [32]. Cells were split twice weekly and plated at a density of 5×10^5^ or 1×10^6^ on 35 and 60 mm plates, respectively. Cells to be differentiated were plated on Growth Factor Reduced BD Matrigel Matrix (BD Biosciences; 1∶75 in DMEM/F12) coated plates. The cells derived in this study may be obtained upon written consent from ViaCyte Inc.

### DNA constructs

Transcriptional Activator Like Effector Nucleases (TALEN)s were generated in house using the TALE toolbox (pTALEN_v2) [33]. Guanine binding was encoded by the repeat-variable diresidue Asn-His (NH) as described [34]. TALEN binding sites flanked the stop codon of the *OCT4* gene with the forward TALEN designed to bind to the sequence: 5′- TCTGGGCTCTCCCATGCATT-3′ and the reverse TALEN to the sequence: 5′- TCCCCCATTCCTAGAAGGGC-3′.

The CRISPR/Cas vector was based on px458 (Addgene; plasmid 48138)); however, the Cbh promoter was exchanged for a full-length CAGGS promoter in order to maximize hESC expression (pCCC). The gRNA (AGAGTGGTGACGGAGACAGG; score 0.6) was designed using the algorithm reported by Doench *et al*. [35] and was cloned into the BbsI sites of pCCC to generate pCCC-LL488 as described by Ran *et al*. [36]. The targeting vector was obtained from Addgene (plasmid 31939) and has been previously described [21].

### Electroporation

CyT49 hESC were cultured in 10/10 media with 1 µM Y-27632 dihydrochloride (Tocris Bioscience) for 2 hours prior to electroporation. Cells were washed with PBS before trypsinization with Accutase (Life Technologies) for 5 minutes at 37°C. Following detachment, cells were centrifuged at 200×g for 5 minutes before being washed three times in 100-pellet volumes of PBS. 10^7^ cells were resuspended in Embryomax Electroporation Buffer (Millipore), transferred to a 0.4 cm cuvette with 40 µg of OCT4-eGFP-2A-Puro donor plasmid and 15 µg of each TALEN encoding plasmid (or 15 µg pCCC-LL488), and electroporated using Bio-Rad Gene Pulser II system (250 V, 500 µF, time constants <13 ms). After electroporation, cells were resuspended well in 10/10 media with 1 µM Y-27632 dihydrochloride and plated onto a 10 cm Matrigel-coated tissue culture dish. Media was replaced daily with 10/10 and cells were allowed to recover for up to four days before selecting with 0.25 µg/mL puromycin (Sigma). Colonies were picked into a 96-well Matrigel-coated plate within 10 days of electroporation by manually scraping and pipetting the colony off the plate and into a well with 100 uL of 10/10. Once clones were close to confluent, cells were replica plated onto three plates: one to genotype, one to freeze down and one to expand the correctly targeted clones. Genomic DNA was extracted using QuickExtract (Epicentre) and the following primers were used to genotype: 5′F CTCAGTTCTGCTGGGATAAG, 5′R GTCTTGTAGTTGCCGTCGTC, 3′F GCAACCTCCCCTTCTACGAG, 3′R CTTACACCAAGCCAAACTATTG.

### *In* *vitro* differentiation of hESC

The differentiation protocol was adapted from Schulz *et al*. [32]. Briefly, to produce definitive endoderm cells were treated with Activin A (100 ng/mL; eBioscience), Wnt3a (25 ng/mL; R&D), and 1∶5000 Insulin-Transferrin-Selenium (ITS; Gibco) in RPMI (0.5x penicillin/streptomycin, 1x glutaMAX; Hyclone) for 24 hours. Cells were in the same media supplemented with 0.2% defined FBS and without Wnt3a for another 48 hours. To generate primitive gut tube, cells were first treated with KGF (25 ng/mL; R&D), TGF-β RI kinase inhibitor (2.5 µM; EMB Bioscience), 0.2% defined FBS and 1∶1000 ITS. Cells were then treated as previously but without TGF-β RI kinase inhibitor for 48 hours. To produce posterior foregut, cells were treated for 36 hours in TTNPB (3 nM; Sigma), cyclopamine-KAAD (0.25 µM; Toronto Research Chemicals), Noggin (50 ng/mL; R&D), 0.5x B27 (Gibco) in DMEM High Glucose (0.5x penicillin/streptomycin, 1x glutaMAX; Hyclone). Finally, to produce pancreatic progenitors and endocrine precursors, cells were treated for 36 hours in Noggin (50 ng/mL; R&D), KGF (50 ng/mL; R&D), EGF (50 ng/mL; R&D) in DMEM High Glucose.

### RNA isolation and RT-PCR analysis

RNA extraction was performed as previously described [37]. Gene expression analysis was determined using ΔΔCT relative to the housekeeping gene, TATA-binding protein (TBP). For a list of TAQMAN primers used, see Table S1.

### Western blot analysis

Lysis buffer (95°C) was used to lyse cells and protein was denatured by boiling at 95°C for 10 minutes before sonication (S-4000 with cuphorn; Misonix) for 2 minutes (80%). Cells were then centrifuged at 10,000×g for 5 minutes at 20°C and supernatant was collected. Lysates were subjected to standard SDS-PAGE followed by blotting onto nitrocellulose membrane (Biorad). Blots were then blocked with 5% milk powder in Tris-buffered saline with Tween (0.1%) and probed with rabbit anti-human OCT4 (Cell Signaling; 1∶1000), anti-GFP (MBL; 1∶1000) or mouse anti-GAPDH (Sigma; 1∶125,000) overnight at 4°C in blocking buffer. The next day membranes were probed with horseradish peroxidase-conjugated secondary antibodies at 1∶10,000 (Jackson ImmunoResearch) for 1 hour and visualized with ECL Prime (GE Biosciences).

### Flow Cytometry and FACS

Cells were rinsed once with PBS and detached from the plate using Accutase. Cells were centrifuged for 5 minutes at 200×g before being resuspended well in 4% paraformaldehyde (PFA) and fixed for 15 minutes. Subsequently, cells were rinsed twice in PBS before analyses on a BD FACSCalibur flow cytometer for GFP expression. Representative flow plots can be found in Figure S4B. To sort for eGFP^+^ and GFP^–^ populations, cells were trypsinized, washed in PBS and sorted directly into TRIzol (Life Technologies) using a BD FACS Aria. To determine endogenous OCT4 expression, cells were fixed as above, permeabilized in 0.5% triton-X and stained overnight at 4° with rabbit anti-human OCT4 antibody (1∶100; Cell Signaling). The next morning cells were rinsed three times in PBS before incubation with secondary antibody anti-rabbit FITC (1∶250; Jackson ImmunoResearch) for 1 hour at room temperature. Cells were analyzed on a BD FACSCalibur using appropriate unstained and secondary antibody only controls. Representative flow plots for OCT4 analysis can be found in Figure S4A. To determine CXCR4+ cells, cells were fixed in 4% PFA for 15 minutes, washed three times in PBS and incubated with CXCR4-PE antibody (1∶20; R&D) for 45 minutes. After cells were washed well, they were analyzed using BD FACSCalibur.

### Immunocytochemical analyses

Cells were grown and differentiated on Matrigel-coated optical dishes (MatTek). On the day of collection, cells were rinsed once in PBS before fixation in 4% PFA for 15 minutes. Cells were permeabilized with 0.5% triton X in PBS for 30 minutes, blocked for 30 minutes in 5% horse serum in PBS and stained with primary antibodies overnight at 4°C: mouse anti-OCT4 antibody (1∶100; Cell Signaling), mouse anti-SOX2 (1∶100; Cell Signaling), mouse anti-NANOG (1∶100; Cell Signaling), rabbit anti-SOX9 (1∶500; Millipore), and mouse anti-NKX6.1 (1∶100; DSHB). The following morning, plates were washed three times with PBS and stained with secondary antibodies for 1 hour: anti-mouse Dy-488 (1∶250; Jackson ImmunoResearch), anti-mouse Dy-594 (1∶450; Jackson ImmunoResearch), anti-rabbit Dy-594 (1∶450; Jackson ImmunoResearch), and TO-PRO Iodide (1∶10,000; Life Technologies). Images were taken using 63x oil immersion objective on a Leica TCS SP8 confocal microscope.

### Statistical analyses

Statistical analyses were performed using Prism 5 (GraphPad Software). All data are presented as mean ± s.e.m. Data were analyzed using either a Student’s *t* test or a one-way ANOVA with a Dunnett post-hoc test. Significance was determined using p<0.05.

## Results

### Generation of OCT4-eGFP-2A-Puro hESC lines using genetically engineered nucleases

**Figure 1:**
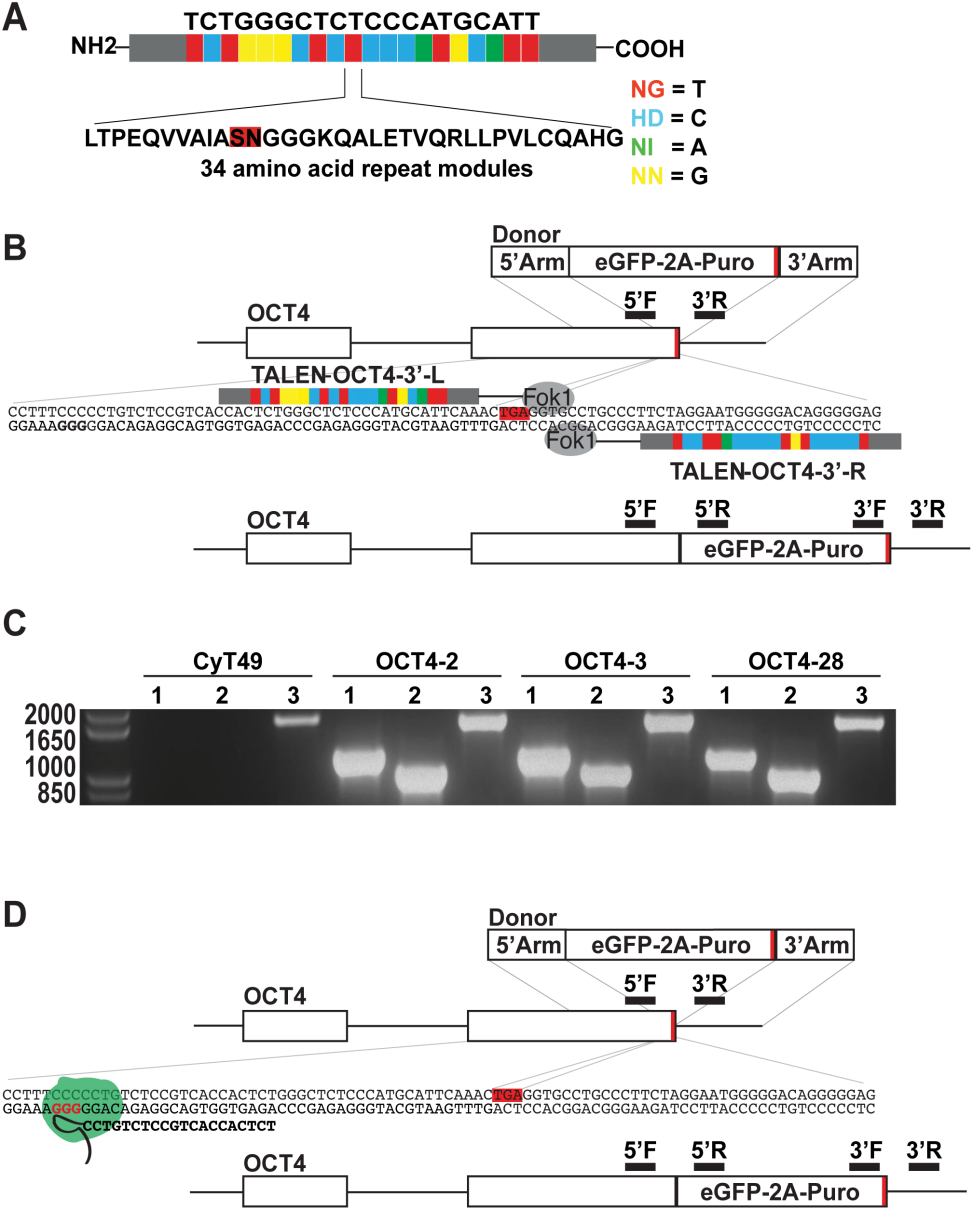
Targeting strategy using genetically engineered nucleases to generate OCT4-eGFP-2A-Puro hESC lines. (A) The structure of Xanthomonas sp TALE protein. Each nucleotide-binding module is comprised of a 34 amino acid sequence, inside of which is embedded one of 4 repeat variable domains (RVD). The sequence of this di-amino acid RVD dictates the deoxynucleotide-binding cipher: NG is highly specific for deoxythymidine, HD for deoxycytidine, NI for deoxyadenosine, and NH for deoxyguanosine. (B) Schematic overview of the targeting strategy using TALENs to knock eGFP onto the OCT4 coding sequence. Red line represents the stop codon. Regions where genotyping PCR primer pairs bind are highlighted for 5′F, 5′R, 3′F and 3′R. (C) Genotyping PCR for i) 5′ arm of insertion using primers 5′F and 5′R (1180 bp) ii) 3′ arm of insertion using primers 3′F and 3′R (1000 bp) iii) endogenous allele using primers 5′F and 3′R for parent CyT49 line and three of the generated knock-in lines OCT4-2, OCT4-3 and OCT4-28. (D) Schematic overview of the CRISPR-Cas9 targeting strategy. Red line represents the stop codon. A green circle represents the Cas9 endonuclease with the tracRNA in black. The genomic protospacer adjacent motif (PAM) sequence is highlighted in red type and the guide RNA sequence is in bold type. Genotyping PCR primer pairs are the same as for TALEN targeting and are highlighted.

TALENs consist of 34 amino acid repeat modules where the repeat variable domain at amino acids 12–13 dictates nucleotide binding specificity (Figure 1A) [38], [39]. Using this modular code, a TALEN pair was designed that bound on either side of the *OCT4* stop codon where the Fok1 nuclease domains of this pair of proteins homodimerize and generate a double stranded break (DSB) (Figure 1B). This DSB can be repaired through homologous recombination of the provided donor plasmid, resulting in eGFP-2A-Puro inserted, in frame, downstream of the last exon of *OCT4*.

Applying this strategy, 52 puromycin-resistant clones from two electroporations were picked and characterized. Sixteen of these clones (31%) were correctly targeted at both the 5′ and 3′ ends as determined using PCR genotyping (Table 1). As the primer pairs used to amplify the 5′ and 3′ regions of the genomic insertion contained one primer that bound outside of OCT4 donor vector homology arms and a second primer that bound within sequences not contained in wild type cells (Figures 1B & S1B), these experiments correctly distinguished targeted clones from those with random genomic insertions. Furthermore, sequence analyses of the obtained PCR products confirmed precise insertion of the reporter gene (Figure S1) without introduced errors. To determine if the insertion was found in one or both alleles, PCR genotyping was used to distinguish the wild type allele from the modified allele and it was determined that all three hESC lines were heterozygous for the insertion (Figure 1C). Taken together, these results demonstrate that this new TALEN pair can drive efficient genomic modification downstream of OCT4 in hESCs.

**Table 1:** Targeting efficiency of TALEN/CRISPR-mediated OCT4-eGFP-2A-Puro CyT49 hESC lines.

DONOR PLASMID	DONOR AMOUNT	NUCLEASE (AMOUNT)	CELL NUMBER	NUMBER OF CLONES	TARGETED AT 5′	TARGETED AT 3′	CORRECTLY TARGETED	TARGETING EFFICIENCY (%)
OCT4-eGFP-2A-Puro	40 ug	TALEN (15 ug each)	10 million	52	32	25	16	31
OCT4-eGFP-2A-Puro	40 ug	CRISPR-Cas (15 ug)	10 million	32	31	15	15	47

As OCT4 has not been targeted using the CRISPR-Cas system and previous reports suggest CRISPR-Cas9 is more efficient, we compared the efficiencies of TALEN and CRISPR-Cas9 technologies. CRISPR-Cas9 is an RNA-guided endonuclease technology [22], [40] and requires three distinct components: the guide RNA (gRNA), the tracRNA, and the Cas9 endonuclease. The gRNA binds to target genomic sequences by complementary base pairing and recruits first the tracRNA and then the Cas9 endonuclease (Figure 1D), which creates a DSB that is repaired by the same mechanisms as described above.

Early attempts at targeting the OCT4 locus using the protocol outlined by Ran et al. [36] were unsuccessful, with only one of 177 puro-resistant clones from three separate electroporations correctly targeted at the 5′ end (data not shown). Because of low expression from the Cbh promoter in CyT49 cells, a new CRISPR-Cas9 vector was generated that utilized the full length CAGGS promoter to drive Cas9 expression. Using this expression system, 15/32 (47%) clones were correctly targeted at both the 5′ and 3′ ends (Table 1) and one of these clones was homozygous for the insertion while all others were heterozygous. These data are consistent with other reports [20], [41] that suggest the CRISPR-Cas9 strategy is more efficient than TALENs at generating DSBs.

### OCT4-eGFP reporter lines have normal OCT4 expression and stem cell phenotype

**Figure 2:**
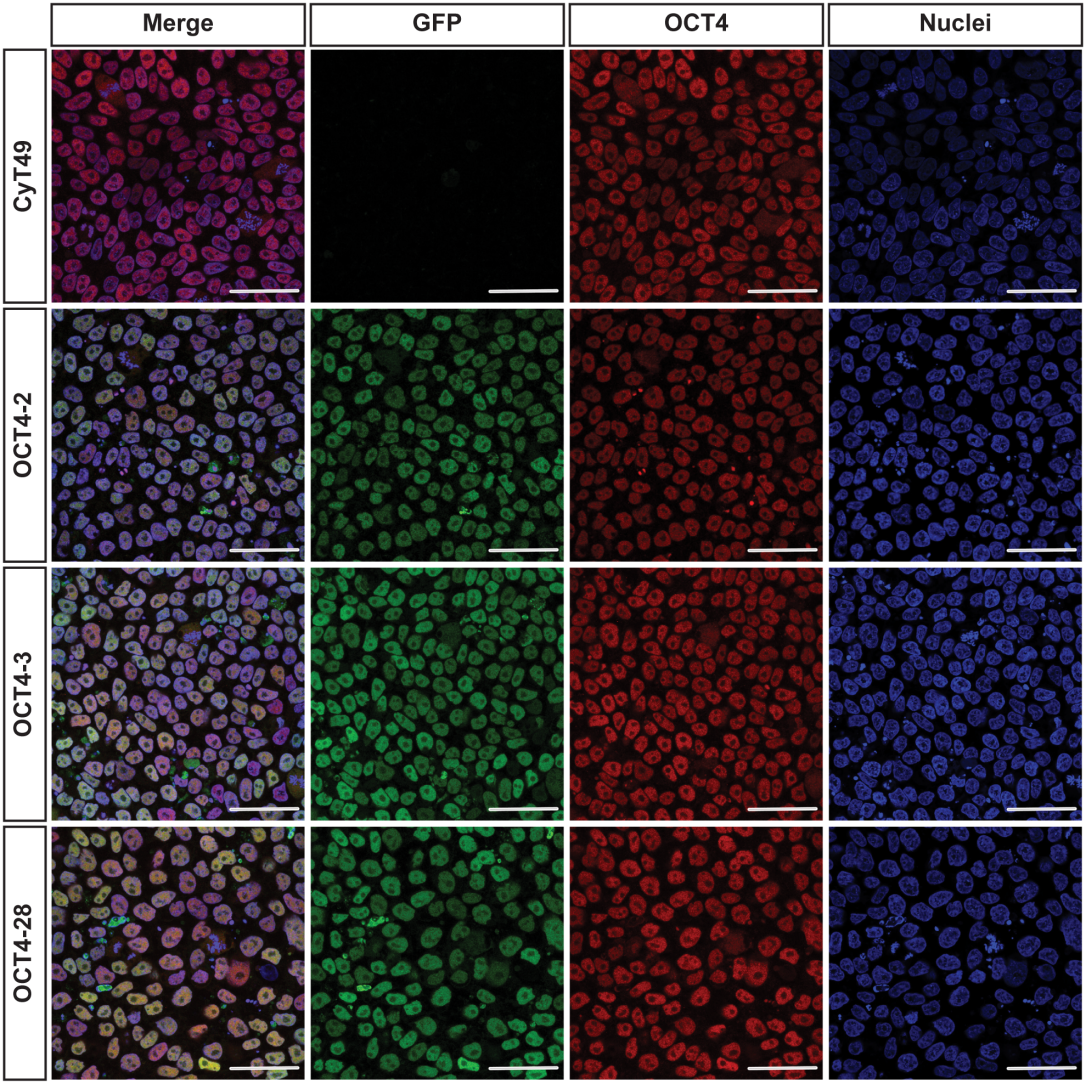
Knock-in add on of eGFP does not impact native OCT4 expression in targeted hESC lines. Undifferentiated cells on optical dishes were fixed in 4% PFA, permeabilized using 0.5% triton-X and stained for OCT4. Images were obtained on a Leica SP8 confocal microscope and native eGFP fluorescence (green) overlapped completely with both OCT4 immunostaining (red) and nuclear counterstain using TO-PRO Iodide (blue). Scale bars represent 50 µm.

To determine if the knock-in add-on eGFP faithfully reported expression of *OCT4*, immunofluorescence for OCT4 in the wildtype CyT49 and the knock-in hESC lines OCT4-2, OCT4-3, and OCT4-28 was performed. As depicted in Figure 2, OCT4 and GFP expression completely overlapped in these three cell lines indicating that GFP faithfully recapitulates endogenous *OCT4* expression. Further, similar OCT4 staining intensities between targeted and parental cells suggested that the targeting did not affect native *OCT4* expression levels. Importantly, all three of the reporter lines maintained eGFP expression after eight passages in culture (data not shown), confirming the stability of this insertion and minimal effects on maintenance of pluripotency.

**Figure 3:**
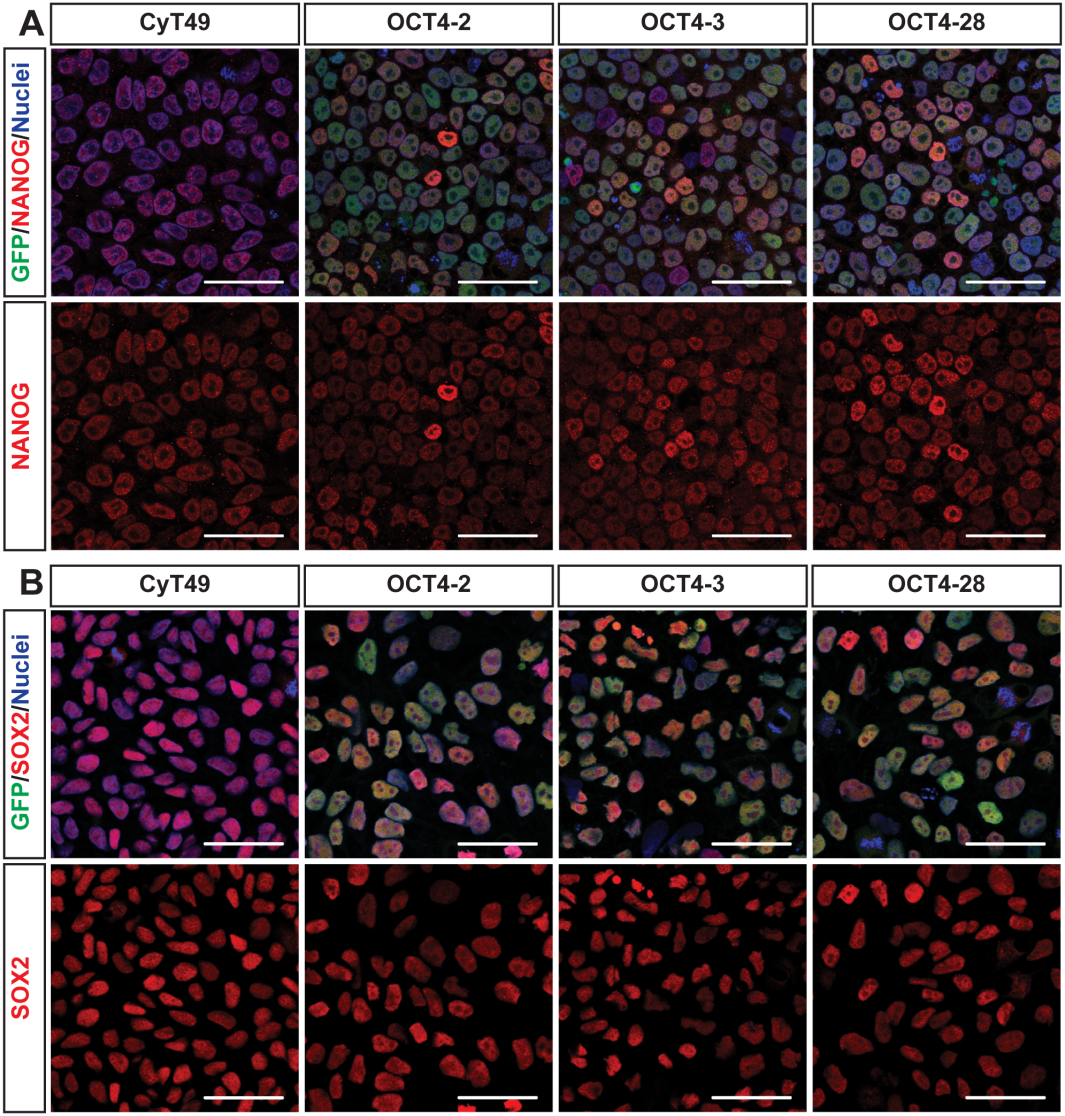
Stem cell characteristics are retained in OCT4-eGFP-2A-Puro reporter hESC lines. Cells were grown on optical dishes and then fixed with 4% PFA and permeabilized with 0.5% triton-X. Immunostaining was carried out and images were obtained on a Leica SP8 confocal microscope. Native eGFP fluorescence (green) overlapped with both SOX2 or NANOG immunostaining (red) and nuclear counterstain using TO-PRO Iodide (blue). Scale bars represent 50 µm.

To ensure the genomic modification and our targeting approach did not alter the stem cell characteristics of these cells, immunofluorescent staining for two other pluripotency markers, NANOG (Figure 3A) and SOX2 (Figure 3B) was performed. As seen in Figure 3, similar immunostaining intensities for these pluripotency factors were observed in all four cell lines, suggesting that the targeting and cloning did not impact pluripotency. Interestingly, a small percentage of SOX2+GFP- and NANOG+GFP- cells in all three OCT4 reporter lines was noted.

### OCT4-eGFP reporter lines are able to form the definitive endoderm germ layer

**Figure 4:**
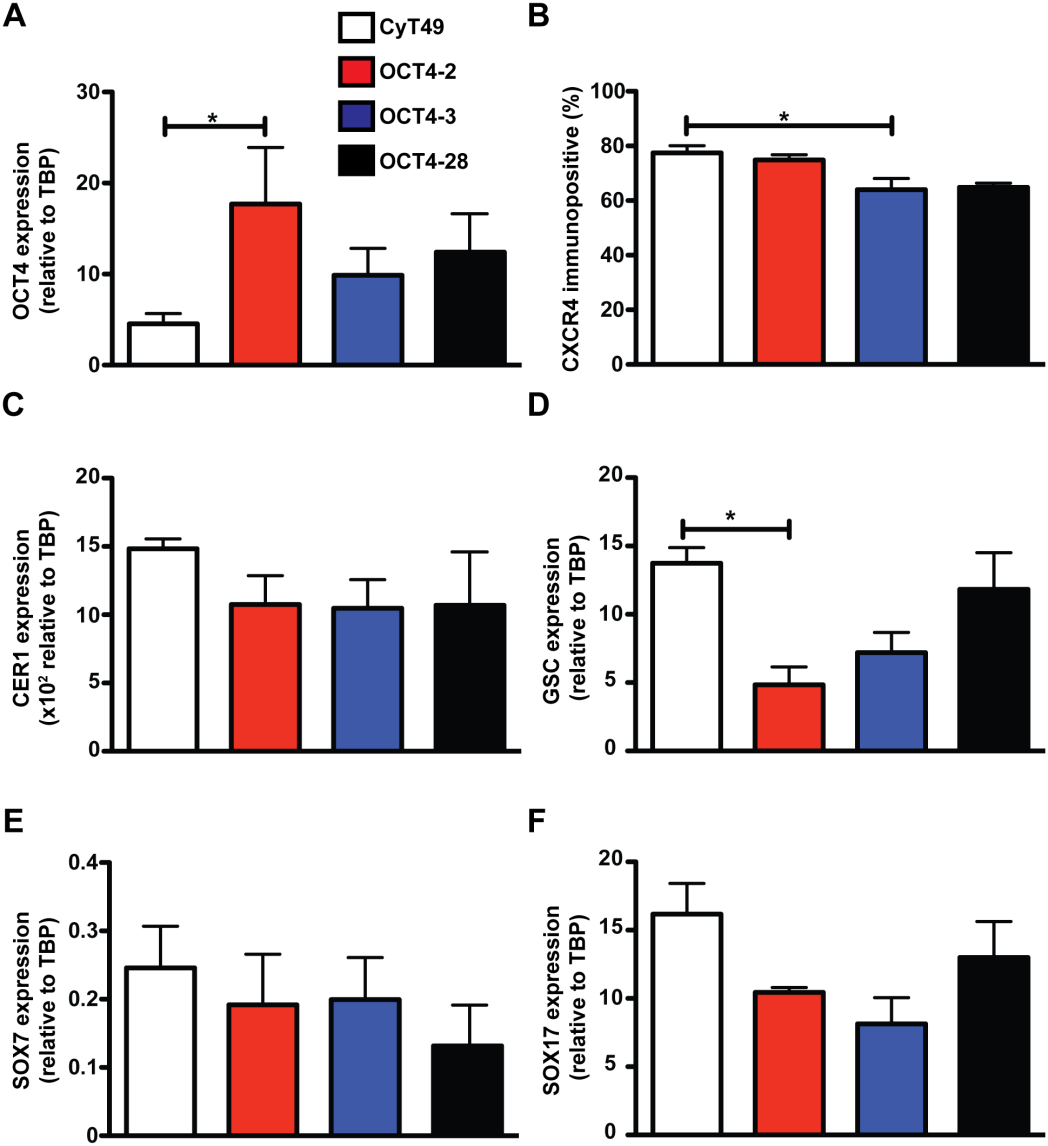
Differentiation of the definitive endoderm germ layer is unaffected by the addition of eGFP into the OCT4 locus. hESCs were differentiated into definitive endoderm using a three day protocol, cells were collected and expression of OCT4 (A), CER1 (C), GSC (D), SOX7 (E), and SOX17 (F) were analyzed using Taqman qPCR. All genes were normalized to TATA Binding Protein expression (TBP). (B) hESC-derived DE cells were fixed with 4% PFA and stained for the cell surface marker CXCR4. The number of CXCR4+ DE cells was detected using BD FACSCalibur in the CyT49, OCT4-2, OCT4-3 and OCT4-28 lines. Statistical analysis was carried out using a one-way ANOVA followed by a Dunnett post-test. n≥3. *p<0.05.

To determine if the genomic modification impacted differentiation of these cells and if *OCT4* expression levels were downregulated at similar rates during definitive endoderm (DE) formation in targeted lines, a directed differentiation to DE and qPCR was carried out. During DE formation, only the OCT4-2 line showed significantly elevated levels of OCT4 when compared to CyT49 controls (Figure 4A). Next, the efficiency of DE formation was determined by measuring the number of CXCR4 immunopositive(+) cells using flow cytometry [42]. As seen in Figure 4B, there were no differences in the number of CXCR4+ cells derived from the OCT4-2 or OCT4-28 lines; however, there was a slight, 13% reduction in CXCR4+ cells in the OCT4-3 line. The OCT4-eGFP lines expressed normal message levels of DE markers including: CER1 (Figure 4C), GSC (Figure 4D), and SOX17 (Figure 4F). Of note, none of these markers were significantly different between the reporter lines and CyT49, except for a 2.8-fold reduction in GSC message expression again in OCT4-2. *SOX7* expression was measured to determine if there was any change in the formation of visceral endoderm (VE) (Figure 4E) and no significant differences were observed. Taken together, these results suggest that knocking eGFP onto *OCT4* has minimal effects on the formation of DE beyond the previously described clonal variation [43].

To demonstrate the utility of eGFP to enrich for *OCT4* expression, the reporter lines were differentiated to definitive endoderm, FACS was used to collect the eGFP+ and eGFP- cells, and OCT4 qPCR was carried out on the two populations. As expected, *OCT4* expression was enriched 2.7-, 1.5-, and 4.4-fold in the eGFP+ cells from OCT4-2, OCT4-3, and OCT4-28 lines, respectively (Figure S2). Surprisingly, abundant OCT4 mRNA was observed in the GFP- fraction of OCT4-3. As SOX17 is important in hESCs for DE formation [44], *SOX17* expression was measured and found to be enriched 19.3-, 118.1-, and 83.3-fold in the eGFP^−^ cells from OCT4-2, OCT4-3, and OCT4-28 lines, respectively (Figure S2). This provides evidence that reporter expression driven by a single endogenous promoter is sufficient to isolate cells via FACS and that these cell lines could be used to assess for presence of *OCT4*-expressing cells in mixed, differentiated cultures.

### eGFP expression mirrors OCT4 protein levels in OCT4 reporter lines

**Figure 5:**
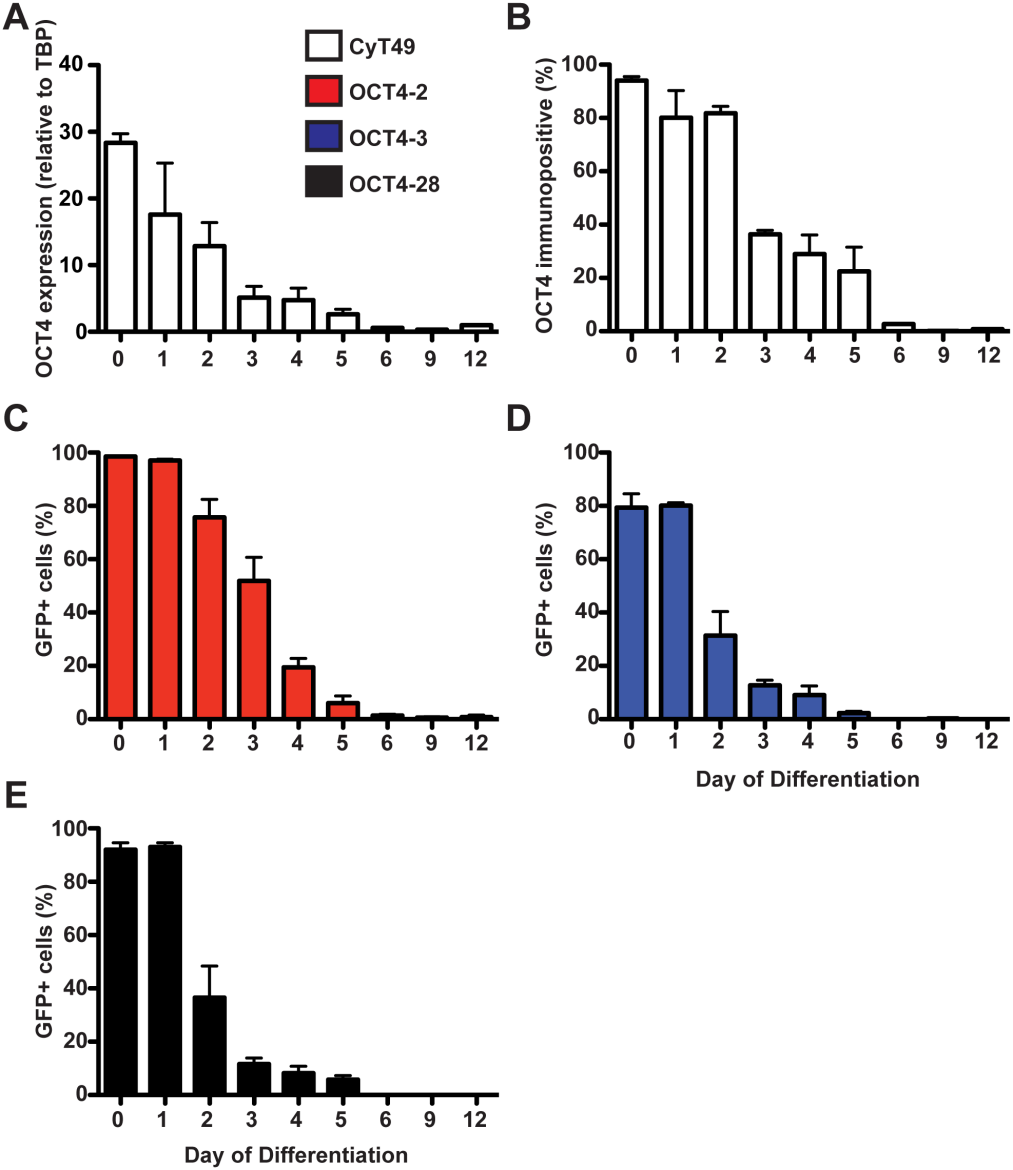
GFP expression decreases upon differentiation towards pancreatic progenitor cells. (A) The mRNA expression of OCT4 was measured during the differentiation of CyT49 cells. Day 0 represents undifferentiated hESC cells, Days 1–3 are cells becoming definitive endoderm, Days 4–6 are cells becoming posterior foregut, Day 9 are pancreatic endoderm cells and Day 12 are pancreatic progenitors and endocrine cells. (B) CyT49 cells were fixed with 4% PFA, permeabilized in 0.5% triton-X, stained using rabbit anti-OCT4 antibodies followed by FITC-conjugated donkey anti-rabbit antibodies. The number of FITC+ cells was measured using a BD FACSCalibur during several days of the differentiation protocol. The number of GFP^+^ cells was measured using native eGFP fluorescence and a BD FACSCalibur in OCT4-2 (C), OCT4-3 (D) and OCT4-28 (E) fixed cells. n≥3.

To determine whether the stability of the OCT4-eGFP fusion protein is similar to that of native OCT4, western blot analyses of OCT4 and GFP in stem cells (Day 0), definitive endoderm (Days 1–3) and posterior foregut (Days 4–6) was performed (Figure S3). The decline of OCT4 protein in the CyT49 parental line was consistent with mRNA expression (*c.f.* Figure 5A & Figure S3). Furthermore, the OCT4-eGFP fusion protein was downregulated at a similar rate to wild type OCT4 in all three genetically modified reporter lines and the rate of OCT4 loss is consistent with the FACS data (Figure 5B).

### Ability to differentiate into pancreatic progenitors is maintained in OCT4 reporter lines

In order to determine whether fusion protein would alter OCT4 stability and possibly the dynamics of differentiation towards endodermally-derived tissues, *OCT4* gene expression (Figure 5A) and the number of OCT4+ cells (Figure 5B) was assessed during the differentiation towards pancreas. To confirm that the loss of eGFP expression in the reporter lines mirrors the decline in OCT4 expression that is observed in the parental line (Figures 5A&B), we characterized eGFP expression during the differentiation towards pancreas in the OCT4 reporter lines using flow cytometry (Figures 5C–E). In undifferentiated cells, 98.6%, 79.3%, and 92.1% of cells were GFP+ in OCT4-2, OCT4-3, and OCT4-28 lines, respectively. In human pluripotent cells, OCT4 interacts with both NANOG and SOX2 to activate pluripotent genes [45]; however, it has been shown that elevated *Oct4* expression is sufficient for endodermal formation in mouse ESCs [46] due to the cooperation between Oct4 and Sox17 in driving endoderm differentiation [28]. Consistent with the role of OCT4 in endoderm formation we noted 51.9%, 12.7%, and 11.7% GFP+ cells at the end of day 3 in OCT-2, OCT4-3, and OCT4-28 lines, respectively. Furthermore, we did not detect greater than 1% GFP+ in hESC-derived pancreatic endoderm at day 12 from any of the reporter lines, which is consistent with the number of OCT4+ cells in the CyT49 line (Figures 5A&B). Thus, in agreement with data presented in Figure 4A and Figure S3, OCT4-2 has a delayed loss of OCT4 protein upon differentiation, which may result in a delayed formation of endodermally-derived progenitors.

**Figure 6:**
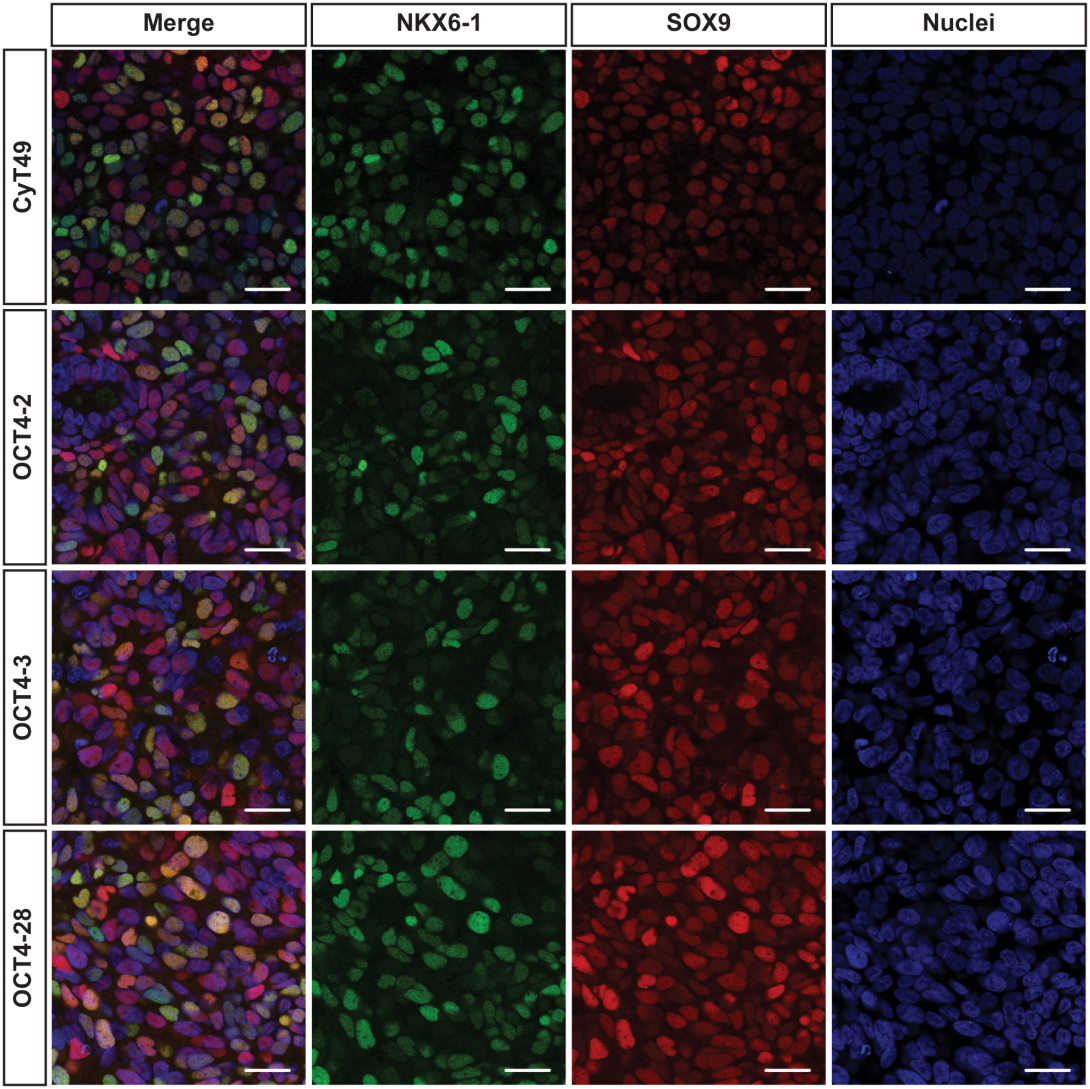
Addition of eGFP does not affect the efficiency of pancreatic progenitor formation during *in vitro* differentiation protocol. Immunostaining for NKX6-1 (green) and SOX9 (red) was carried out on day 12 in parent CyT49 hESCs and knock-in OCT4-2, OCT4-3 and OCT4-28 hESC lines that were grown and differentiated in optical dishes. Images were obtained on a Leica SP8 confocal microscope using TO-PRO Iodide (blue) as nuclear counterstain. Scale bars represent 25 µm.

To determine whether the targeted clones would differentiate towards the pancreatic lineage with similar efficiencies as the parental line, immunocytochemical analyses for NKX6-1 [47]–[50] and SOX9 [51]–[54] were performed on day 12 (Figure 6). Despite the small differences in loss of *OCT4* expression described above, we were unable to appreciate a change in the level of these two proteins in the reporter lines compared to the parental line. As NKX6-1 and SOX9 are also expressed in other cell types, qPCR analysis for other pancreas and endocrine cell genes was carried out. No significant differences in expression levels of *PDX1*, *NEUROG3*, *SOX9* or *NKX6-1* were observed (not shown). Taken together, these findings suggest that this approach is amenable for the future creation of tissue or cell-type specific reporter lines.

## Discussion

This study outlines two strategies for the creation of OCT4-eGFP-2A-Puro hESC reporter lines using either TALEN or CRISPR-Cas9 genome editing methodologies. Both of these approaches allowed for the efficient generation of reporter lines in approximately four weeks. Further characterization of these lines determined that knocking a fluorescent protein onto *OCT4* neither impacted the pluripotency nor differentiation potential of the cells. We confirmed that the eGFP reporter is co-expressed with OCT4 and does not alter native OCT4 expression. Finally, the efficiencies of differentiating these cells to both definitive endoderm and pancreatic progenitors were similar to the parental CyT49 line.

Genome-editing technologies such as those described herein have greatly improved efficiency of homologous recombination in three ways: 1) through reducing the burden of generating constructs containing long homology arms; 2) by simplifying clone screening and verification processes; and 3) by increasing the likelihood of homologous recombination. Two other studies have shown the utility of genome editing in generating mutations at the *OCT4* locus; however, neither of these studies have characterized whether these mutations impact differentiation potential. Zinc Finger Nucleases were used to insert eGFP into one of two regions of *OCT4*, both of which disrupted the protein coding region in BG01 hESCs [20]. More recently, using TALENs, eGFP was inserted downstream of the last exon of *OCT4* to create an OCT4-eGFP fusion protein, avoiding the disruption of the protein coding gene [21]. This manuscript provides the first description *OCT4* modification using the CRISPR-Cas9 system. Using the described approaches, the CRISPR-Cas9 strategy was more efficient (47% vs. 31%) and allowed creation of homozygous knock-in reporter lines.

OCT4, a POU domain transcription factor that can act both as an activator and repressor, was first identified as a central member of the pluripotency network [24], [55]. As such, its absence causes ESCs to differentiate into trophoblast cells [46]. Importantly no changes in the expression of NANOG or SOX2 were observed in the targeted cell lines, consistent with the fact that the OCT4-eGFP fusion protein did not alter the pluripotent nature of these hESC lines. However, *OCT4* expression was observed in the GFP- fraction from OCT4-3 line. This could reflect a mixed clone and highlights a limitation of this approach, which is that ensuring lines are derived from a single cell can often be difficult. As such, it should be emphasized that it is important to investigate several “clonal” lines when performing these types of analyses.

OCT4 is required for the generation of all germ layers [25] and in particular definitive endoderm (DE) [28], the germ layer whose derivatives form the lining of the gut and associated organs [56]. In the three OCT4-eGFP reporter lines characterized here, there were no significant differences in expression of the DE genes *CER1* and *SOX17* and the VE gene *SOX7*; however, there were significant changes in *GSC* in OCT4-2. *GSC* is a homeobox gene with important roles in gastrulation and endoderm formation. Interestingly, the significant decrease in *GSC* expression was concurrent with a significant increase in *OCT4* expression. Owing to its role as a transcriptional repressor [57], it is possible that *GSC* expression, and endoderm formation, is repressed by prolonged expression of OCT4. Even with the slight differences in the expression profile of the DE generated from these reporter lines, they were able to form pancreatic progenitors with similar efficiencies, suggesting that this strategy does not dramatically disrupt differentiation.

In summary, this work has demonstrated that the CyT49 hESC line is amenable to genomic modification using two genome-editing technologies. Characterization of three OCT4-eGFP reporter lines demonstrated that these genomic modifications do not significantly alter either their stem cell characteristics or differentiation potential. These studies add to the growing body of literature that shows nuclease-mediated genome engineering is a powerful approach for hESC modification and underscore its utility in the generation of personalized cell based therapies.

## Supporting Information

Figure S1 Sequencing analysis of OCT4-2, OCT4-3, and OCT4-28 hESC clones. Genomic DNA isolated from each cell line was sequenced using primers 5′F, 5′R, 3′F, and 3′R. (A) Aligned raw sequences from OCT4-2, OCT4-3, and OCT4-28 spanning homology arms. (B) Schematic of pOCT4-eGFP-2A-Puro genomic integration outlining the 5′ homology arm (pink box), eGFP (green box) and 3′ homology arm (pink box). Arrows highlight region of homology arm sequence alignment for OCT4-2 (red), OCT4-3 (blue) and OCT4-28 (black). Forward primers (hatched arrows) and reverse (solid arrows) genotyping and sequencing primers are highlighted.
(PDF)

Figure S2 OCT4 and SOX17 expression in GFP^+^ and GFP^−^ cells. Cells were trypsinized on the second day of differentiation to definitive endoderm and the GFP^+^ and GFP^−^ populations were collected into TRIzol using the BD FACS Aria. RNA was isolated and cDNA synthesized before carrying out qPCR analysis for OCT4 and SOX17 using TBP as control gene. Statistical analysis was performed using a Student’s *t*-test. n≥3. *p<0.05, **p<0.01, ***p<0.001.
(TIF)

Figure S3 Western Blot analysis of OCT4 and eGFP expression during differentiation of hESCs to definitive endoderm and primitive gut tube. Protein lysates were collected on Day 0 (hESC), Days 1–3 (definitive endoderm) and Days 4–6 (primitive gut tube) and the expression of OCT4, eGFP and the control protein GAPDH were analyzed in the CyT49, OCT4-2, OCT4-3 and OCT4-28 hESC lines using SDS-PAGE followed by western blotting as described in the Materials and Methods section.
(TIF)

Figure S4 Representative flow cytometry data for the analysis of OCT4^+^ and eGFP^+^ cells. (A) Representative flow plots for CyT49 cells that were collected on Days 0–3, stained for OCT4, and analyzed using BD FACSCalibur. Unstained controls used to set up the gating strategy are also shown. (B) Representative flow plots for OCT4-2 cells that were analyzed for eGFP expression using the BD FACSCalibur on Days 0–3. To set up the gates for eGFP, CyT49 cells were used as a negative control. Data analysis was performed using FlowJo software.
(TIF)

Table S1 List of Taqman qPCR primers.
(PDF)
