# Supplementary material for: TALEN/CRISPR-Mediated eGFP Knock-In Add-On at the OCT4 Locus Does Not Impact Differentiation of Human Embryonic Stem Cells towards Endoderm
Source: PLoS One. 2014 Dec 4;9(12):e114275. doi: 10.1371/journal.pone.0114275 (PMC4256397; doi:10.1371/journal.pone.0114275)

```
                      10        20        30        40        50        60        70        80        90       100
pOCT4-eGFP-2A-TCTTGGCCTAATTTCCATTGCTTCCATCACTGGCTCGTAGCTCTCCGTCTTTGGTGCAGTGGTTCTCAGTGGGATGGAGTGAAATTCCTCAGTTCTGCTG
              ~~~~~~~~~~~~~~~~~~~~~~~~~~~~~~~~~~~~~~~~~~~~~~~~~~~~~~~~~~~~~~~~~~~~~~~~~~~~~~~~~~~~~~~~~~~~~~~~
Consensus:
              ~~~~~~~~~~~~~~~~~~~~~~~~~~~~~~~~~~~~~~~~~~~~~~~~~~~~~~~~~~~~~~~~~~~~~~~~~~~~~~~~~~~~~~~~~~~~~~~~
Clone 2c.ab1
Clone 3c.ab1
Clone 28c.ab1
Clone 28d.ab1
Clone 2d.ab1
Clone 3d.ab1
Clone 3b.ab1
Clone 2b.ab1
Clone 28b.ab1
Clone 2a.ab1
Clone 3a.ab1
Clone 28a.ab1

                     110       120       130       140       150       160       170       180       190
pOCT4-eGFP-2A-GGATAAGGTCCAGAGCCAACCCTTCCAGGATCCTGCCTTTTCACACCACCACCTGGCTCTGCTGACACATC-TAGTCACAGACCCCTGTGATGCTGTTAC
              ~~~~~~~~~~~~~~~~~~~~~~~~~~~~~~~~~~~~~~~~~~~~~~~~~~~~~~~~~~~~~~~~~~~~~~~~~~~~~~~~~~~~~~~~~~~~~~~~
Consensus:                        CCTT-CA-G-TACTGCCTTTTCACACCACCACCTGGCTCTGCTGACACATC-TAGTCACAGACCCCTGTGATGCTGTTAC
              ~~~~~~~~~~~~~~~~~~~~~~~~~~~~~~~~~~~~~~~~~~~~~~~~~~~~~~~~~~~~~~~~~~~~~~~~~~~~~~~~~~~~~~~~~~~~~~~~
Clone 2c.ab1                    gaCCTT-CA-G-TACTGCCTTTTCACACCACCACCTGGCTCTGCTGACACATC-TAGTCACAGACCCCTGTGATGCTGTTAC
Clone 3c.ab1                     gaatccaagggcaCTGGCTTTTCACACCACCACCTGGCTCTGCTGACACATCATAGTCACAGACCCCTGTGATGCTGTTAC
Clone 28c.ab1                     ccaccatcagcaCTGCCTTTTCACACCACCACCTGGCTCTGCTGACACATC-TAGTCACAGACCCCTGTGATGCTGTTAC
Clone 28d.ab1
Clone 2d.ab1
Clone 3d.ab1
Clone 3b.ab1
Clone 2b.ab1
Clone 28b.ab1
Clone 2a.ab1
Clone 3a.ab1
Clone 28a.ab1

            200       210       220       230          240         250         260         270        280
pOCT4-eGFP-2A-TCAGCAAGTCCAAAGCTTGCCCTTGTCACCCCCTT-CCC-A-CCT-GCAC-AGAT-ATGCAAA-GCAGAAA-CCCT-CGTGCAGG-CCCGAAAG-AGAAA
              ~~~~~~~~~~~~~~~~~~~~~~~~~~~~~~~~~~~~~~~~~~~~~~~~~~~~~~~~~~~~~~~~~~~~~~~~~~~~~~~~~~~~~~~~~~~~~~~~
Consensus:    TCAGCAAGTCCAAAGCTTGCCCTTGTCACCCCCTT-CCC-A-CCT-GCAC-AGAT-ATGCAAA-GCAGAAA-CCCT-CGTGCAGG-CCCGAAAG-AGAAA
              ~~~~~~~~~~~~~~~~~~~~~~~~~~~~~~~~~~~~~~~~~~~~~~~~~~~~~~~~~~~~~~~~~~~~~~~~~~~~~~~~~~~~~~~~~~~~~~~~
Clone 2c.ab1  TCAGCAAGTCCAAAGCTTGCCCTTGTCACCCCCTT-CCC-A-CCT-GCAC-AGAT-ATGCAAA-GCAGAAA-CCCT-CGTGCAGG-CCCGAAAG-AGAAA
Clone 3c.ab1  TCAGCAAGTCCAAAGCTTGCCCTTGTCACCCCCTT-CCC-A-CCT-GCAC-AGAT-ATGCAAA-GCAGAAA-CCCT-CGTGCAGG-CCCGAAAG-AGAAA
Clone 28c.ab1 TCAGCAAGTCCAAAGCTTGCCCTTGTCACCCCCTT-CCC-A-CCT-GCAC-AGAT-ATGCAAA-GCAGAAA-CCCT-CGTGCAGG-CCCGAAAG-AGAAA
Clone 28d.ab1                      gtcacccCCCCCTT-CCCCA-CCTTGCAC-AGAT-ATGCAAAAGCAGAAA-ACCTTCGTGCAGGGCCCGAAAG-AGAAA
Clone 2d.ab1                            gagcgcgTTCCCC-A-CTG-GCACAAGAT-ATGCAAA-GCAGGAA-ACCT-CGTGCAAG-CCCGGAAGGAG-AA
Clone 3d.ab1                                 ttcc-CCC-ACCCT-GCAC-AGATTATGCAAAAGCAGAAAACCCT-CGTGCAGGGCCCGAAAG-AGAAA
Clone 3b.ab1
Clone 2b.ab1
Clone 28b.ab1
Clone 2a.ab1
Clone 3a.ab1
Clone 28a.ab1

             290        300       310       320       330        340        350        360       370       380
pOCT4-eGFP-2A-GCGAA-CCAGTATCGAGAACCGAGTGAGAGGCAACCTGGAGAATTT-GTTCCTGCAGTGCCC-GAAACCC-ACACTGCAGCAGATCAGCCACATCGCCCA
              ~~~~~~~~~~~~~~~~~~~~~~~~~~~~~~~~~~~~~~~~~~~~~~~~~~~~~~~~~~~~~~~~~~~~~~~~~~~~~~~~~~~~~~~~~~~~~~~~
Consensus:    GCGAA-CCAGTATCGAGAACCGAGTGAGAGGCAACCTGGAGAATTT-GTTCCTGCAGTGCCC-GAAACCC-ACACTGCAGCAGATCAGCCACATCGCCCA
              ~~~~~~~~~~~~~~~~~~~~~~~~~~~~~~~~~~~~~~~~~~~~~~~~~~~~~~~~~~~~~~~~~~~~~~~~~~~~~~~~~~~~~~~~~~~~~~~~
Clone 2c.ab1  GCGAA-CCAGTATCGAGAACCGAGTGAGAGGCAACCTGGAGAATTT-GTTCCTGCAGTGCCC-GAAACCC-ACACTGCAGCAGATCAGCCACATCGCCCA
Clone 3c.ab1  GCGAA-CCAGTATCGAGAACCGAGTGAGAGGCAACCTGGAGAATTT-GTTCCTGCAGTGCCC-GAAACCC-ACACTGCAGCAGATCAGCCACATCGCCCA
Clone 28c.ab1 GCGAA-CCAGTATCGAGAACCGAGTGAGAGGCAACCTGGAGAATTT-GTTCCTGCAGTGCCC-GAAACCC-ACACTGCAGCAGATCAGCCACATCGCCCA
Clone 28d.ab1 GCGAA-CCAGTATCGAGAACCGAGTGAGAGGCAACCTGGAGAATTTTGTTCCTGCAGTGCCC-GAAACCC-ACACTGCAGCAGATCAGCCACATCGCCCA
Clone 2d.ab1  GCGAAGCCAGTATCGAGAGCCGAGTGAGAGGCAACCTGGAGAATTT-GTTCCTGCAGTGCCC-GAAACCC-ACACTGCAGCAGATCAGCCACATCGCCCA
Clone 3d.ab1  GCGAA-CCAGTATCGAGAACCGAGTGAGAGGCAACCTGGAGAATTT-GTTCCTGCAGTGCCCCGAAACCCCACACTGCAGCAGATCAGCCACATCGCCCA
Clone 3b.ab1
Clone 2b.ab1
Clone 28b.ab1
Clone 2a.ab1
Clone 3a.ab1
Clone 28a.ab1

                 390       400       410       420       430       440       450       460       470       480
pOCT4-eGFP-2A-GCAGCTTGGGCTCGAGAAGGATGTGAGTGCCATGTCTCTCTGCGGGCTCCATCTCTTTCCCCTGTCACCACCTCGCTTTCCCTAGCTCTGGCTCCTCCAA
              ~~~~~~~~~~~~~~~~~~~~~~~~~~~~~~~~~~~~~~~~~~~~~~~~~~~~~~~~~~~~~~~~~~~~~~~~~~~~~~~~~~~~~~~~~~~~~~~~
Consensus:    GCAGCTTGGGCTCGAGAAGGATGTGAGTGCCATGTCTCTCTGCGGGCTCCATCTCTTTCCCCTGTCACCACCTCGCTTTCCCTAGCTCTGGCTCCTCCAA
              ~~~~~~~~~~~~~~~~~~~~~~~~~~~~~~~~~~~~~~~~~~~~~~~~~~~~~~~~~~~~~~~~~~~~~~~~~~~~~~~~~~~~~~~~~~~~~~~~
Clone 2c.ab1  GCAGCTTGGGCTCGAGAAGGATGTGAGTGCCATGTCTCTCTGCGGGCTCCATCTCTTTCCCCTGTCACCACCTCGCTTTCCCTAGCTCTGGCTCCTCCAA
Clone 3c.ab1  GCAGCTTGGGCTCGAGAAGGATGTGAGTGCCATGTCTCTCTGCGGGCTCCATCTCTTTCCCCTGTCACCACCTCGCTTTCCCTAGCTCTGGCTCCTCCAA
Clone 28c.ab1 GCAGCTTGGGCTCGAGAAGGATGTGAGTGCCATGTCTCTCTGCGGGCTCCATCTCTTTCCCCTGTCACCACCTCGCTTTCCCTAGCTCTGGCTCCTCCAA
Clone 28d.ab1 GCAGCTTGGGCTCGAGAAGGATGTGAGTGCCATGTCTCTCTGCGGGCTCCATCTCTTTCCCCTGTCACCACCTCGCTTTCCCTAGCTCTGGCTCCTCCAA
Clone 2d.ab1  GCAGCTTGGGCTCGAGAAGGATGTGAGTGCCATGTCTCTCTGCGGGCTCCATCTCTTTCCCCTGTCACCACCTCGCTTTCCCTAGCTCTGGCTCCTCCAA
Clone 3d.ab1  GCAGCTTGGGCTCGAGAAGGATGTGAGTGCCATGTCTCTCTGCGGGCTCCATCTCTTTCCCCTGTCACCACCTCGCTTTCCCTAGCTCTGGCTCCTCCAA
Clone 3b.ab1
Clone 2b.ab1
Clone 28b.ab1
Clone 2a.ab1
Clone 3a.ab1
Clone 28a.ab1

                 490       500       510       520       530       540       550       560       570       580
pOCT4-eGFP-2A-CTGCTCTAGGGCTGTTGGCTTTGGACAGAATGTCCAAGCAGTCAGGCCTGTCTCAGCTCATTCTCTAATGTCCTCCTCTAACTGCTCTAGGGCTGTTGGC
              ~~~~~~~~~~~~~~~~~~~~~~~~~~~~~~~~~~~~~~~~~~~~~~~~~~~~~~~~~~~~~~~~~~~~~~~~~~~~~~~~~~~~~~~~~~~~~~~~
Consensus:    CTGCTCTAGGGCTGTTGGCTTTGGACAGAATGTCCAAGCAGTCAGGCCTGTCTCAGCTCATTCTCTAATGTCCTCCTCTAACTGCTCTAGGGCTGTTGGC
              ~~~~~~~~~~~~~~~~~~~~~~~~~~~~~~~~~~~~~~~~~~~~~~~~~~~~~~~~~~~~~~~~~~~~~~~~~~~~~~~~~~~~~~~~~~~~~~~~
Clone 2c.ab1  CTGCTCTAGGGCTGTTGGCTTTGGACAGAATGTCCAAGCAGTCAGGCCTGTCTCAGCTCATTCTCTAATGTCCTCCTCTAACTGCTCTAGGGCTGTTGGC
Clone 3c.ab1  CTGCTCTAGGGCTGTTGGCTTTGGACAGAATGTCCAAGCAGTCAGGCCTGTCTCAGCTCATTCTCTAATGTCCTCCTCTAACTGCTCTAGGGCTGTTGGC
Clone 28c.ab1 CTGCTCTAGGGCTGTTGGCTTTGGACAGAATGTCCAAGCAGTCAGGCCTGTCTCAGCTCATTCTCTAATGTCCTCCTCTAACTGCTCTAGGGCTGTTGGC
Clone 28d.ab1 CTGCTCTAGGGCTGTTGGCTTTGGACAGAATGTCCAAGCAGTCAGGCCTGTCTCAGCTCATTCTCTAATGTCCTCCTCTAACTGCTCTAGGGCTGTTGGC
Clone 2d.ab1  CTGCTCTAGGGCTGTTGGCTTTGGACAGAATGTCCAAGCAGTCAGGCCTGTCTCAGCTCATTCTCTAATGTCCTCCTCTAACTGCTCTAGGGCTGTTGGC
Clone 3d.ab1  CTGCTCTAGGGCTGTTGGCTTTGGACAGAATGTCCAAGCAGTCAGGCCTGTCTCAGCTCATTCTCTAATGTCCTCCTCTAACTGCTCTAGGGCTGTTGGC
Clone 3b.ab1
Clone 2b.ab1
Clone 28b.ab1
Clone 2a.ab1
Clone 3a.ab1
Clone 28a.ab1

                 590       600       610       620       630       640       650       660       670       680
```

```
pOCT4-eGFP-2A-TTTGGATAGAATGTCCAAGCAGAGTCAGGCCCGTCTCAGCTCATTGTCTAATGTCATTCTCCTTTCTGTCATTCACTTGCAGGTGGTCCGAGTGTGGTTC
              ~~~~~~~~~~~~~~~~~~~~~~~~~~~~~~~~~~~~~~~~~~~~~~~~~~~~~~~~~~~~~~~~~~~~~~~~~~~~~~~~~~~~~~~~~~~~~~~~~~~~
Consensus:    TTTGGATAGAATGTCCAAGCAGAGTCAGGCCCGTCTCAGCTCATTGTCTAATGTCATTCTCCTTTCTGTCATTCACTTGCAGGTGGTCCGAGTGTGGTTC
              ~~~~~~~~~~~~~~~~~~~~~~~~~~~~~~~~~~~~~~~~~~~~~~~~~~~~~~~~~~~~~~~~~~~~~~~~~~~~~~~~~~~~~~~~~~~~~~~~~~~~
Clone 2c.ab1  TTTGGATAGAATGTCCAAGCAGAGTCAGGCCCGTCTCAGCTCATTGTCTAATGTCATTCTCCTTTCTGTCATTCACTGGCAGGTGGTCCGAGTGTGGTTC
Clone 3c.ab1  TTTGGATAGAATGTCCAAGCAGAGTCAGGCCCGTCTCAGCTCATTGTCTAATGTCATTCTCCTTTCTGTCATTCACTTGCAGGTGGTCCGAGTGTGGTTC
Clone 28c.ab1 TTTGGATAGAATGTCCAAGCAGAGTCAGGCCCGTCTCAGCTCATTGTCTAATGTCATTCTCCTTTCTGTCATTCACTTGCAGGTGGTCCGAGTGTGGTTC
Clone 28d.ab1 TTTGGATAGAATGTCCAAGCAGAGTCAGGCCCGTCTCAGCTCATTGTCTAATGTCATTCTCCTTTCTGTCATTCACTTGCAGGTGGTCCGAGTGTGGTTC
Clone 2d.ab1  TTTGGATAGAATGTCCAAGCAGAGTCAGGCCCGTCTCAGCTCATTGTCTAATGTCATTCTCCTTTCTGTCATTCACTGGCAGGTGGTCCGAGTGTGGTTC
Clone 3d.ab1  TTTGGATAGAATGTCCAAGCAGAGTCAGGCCCGTCTCAGCTCATTGTCTAATGTCATTCTCCTTTCTGTCATTCACTTGCAGGTGGTCCGAGTGTGGTTC
Clone 3b.ab1
Clone 2b.ab1
Clone 28b.ab1
Clone 2a.ab1
Clone 3a.ab1
Clone 28a.ab1

                   690       700       710       720       730       740       750       760       770       780
pOCT4-eGFP-2A-TGTAACCGGCGCCAGAAGGGCAAGCGATCAAGCAGCGACTATGCACAACGAGAGGATTTTGAGGCTGCTGGGTCTCCTTTCTCAGGGGGACCAGTGTCCT
              ~~~~~~~~~~~~~~~~~~~~~~~~~~~~~~~~~~~~~~~~~~~~~~~~~~~~~~~~~~~~~~~~~~~~~~~~~~~~~~~~~~~~~~~~~~~~~~~~~~~~
Consensus:    TGTAACCGGCGCCAGAAGGGCAAGCGATCAAGCAGCGACTATGCACAACGAGAGGATTTTGAGGCTGCTGGGTCTCCTTTCTCAGGGGGACCAGTGTCCT
              ~~~~~~~~~~~~~~~~~~~~~~~~~~~~~~~~~~~~~~~~~~~~~~~~~~~~~~~~~~~~~~~~~~~~~~~~~~~~~~~~~~~~~~~~~~~~~~~~~~~~
Clone 2c.ab1  TGTAACCGGCGCCAGAAGGGCAAGCGATCAAGCAGCGACTATGCACAACGAGAGGATTTTGAGGCTGCTGGGTCTCCTTTCTCAGGGGGACCAGTGTCCT
Clone 3c.ab1  TGTAACCGGCGCCAGAAGGGCAAGCGATCAAGCAGCGACTATGCACAACGAGAGGATTTTGAGGCTGCTGGGTCTCCTTTCTCAGGGGGACCAGTGTCCT
Clone 28c.ab1 TGTAACCGGCGCCAGAAGGGCAAGCGATCAAGCAGCGACTATGCACAACGAGAGGATTTTGAGGCTGCTGGGTCTCCTTTCTCAGGGGGACCAGTGTCCT
Clone 28d.ab1 TGTAACCGGCGCCAGAAGGGCAAGCGATCAAGCAGCGACTATGCACAACGAGAGGATTTTGAGGCTGCTGGGTCTCCTTTCTCAGGGGGACCAGTGTCCT
Clone 2d.ab1  TGTAACCGGCGCCAGAAGGGCAAGCGATCAAGCAGCGACTATGCACAACGAGAGGATTTTGAGGCTGCTGGGTCTCCTTTCTCAGGGGGACCAGTGTCCT
Clone 3d.ab1  TGTAACCGGCGCCAGAAGGGCAAGCGATCAAGCAGCGACTATGCACAACGAGAGGATTTTGAGGCTGCTGGGTCTCCTTTCTCAGGGGGACCAGTGTCCT
Clone 3b.ab1
Clone 2b.ab1
Clone 28b.ab1
Clone 2a.ab1
Clone 3a.ab1
Clone 28a.ab1

                   790       800       810       820       830       840       850       860       870       880
pOCT4-eGFP-2A-TTCCTCTGGCCCCAGGGCCCCATTTTGGTACCCCAGGCTATGGGAGCCCTCACTTCACTGCACTGTACTCCTCGGTCCCTTTCCCTGAGGGGGAAGCCTT
              ~~~~~~~~~~~~~~~~~~~~~~~~~~~~~~~~~~~~~~~~~~~~~~~~~~~~~~~~~~~~~~~~~~~~~~~~~~~~~~~~~~~~~~~~~~~~~~~~~~~~
Consensus:    TTCCTCTGGCCCCAGGGCCCCATTTTGGTACCCCAGGCTATGGGAGCCCTCACTTCACTGCACTGTACTCCTCGGTCCCTTTCCCTGAGGGGGAAGCCTT
              ~~~~~~~~~~~~~~~~~~~~~~~~~~~~~~~~~~~~~~~~~~~~~~~~~~~~~~~~~~~~~~~~~~~~~~~~~~~~~~~~~~~~~~~~~~~~~~~~~~~~
Clone 2c.ab1  TTCCTCTGGCCCCAGGGCCCCATTTTGGTACCCCAGGCTATGGGAGCCCTCACTTCACTGCACTGTACTCCTCGGTCCCTTTCCCTGAGGGGGAAGCCTT
Clone 3c.ab1  TTCCTCTGGCCCCAGGGCCCCATTTTGGTACCCCAGGCTATGGGAGCCCTCACTTCACTGCACTGTACTCCTCGGTCCCTTTCCCTGAGGGGGAAGCCTT
Clone 28c.ab1 TTCCTCTGGCCCCAGGGCCCCATTTTGGTACCCCAGGCTATGGGAGCCCTCACTTCACTGCACTGTACTCCTCGGTCCCTTTCCCTGAGGGGGAAGCCTT
Clone 28d.ab1 TTCCTCTGGCCCCAGGGCCCCATTTTGGTACCCCAGGCTATGGGAGCCCTCACTTCACTGCACTGTACTCCTCGGTCCCTTTCCCTGAGGGGGAAGCCTT
Clone 2d.ab1  TTCCTCTGGCCCCAGGGCCCCATTTTGGTACCCCAGGCTATGGGAGCCCTCACTTCACTGCACTGTACTCCTCGGTCCCTTTCCCTGAGGGGGAAGCCTT
Clone 3d.ab1  TTCCTCTGGCCCCAGGGCCCCATTTTGGTACCCCAGGCTATGGGAGCCCTCACTTCACTGCACTGTACTCCTCGGTCCCTTTCCCTGAGGGGGAAGCCTT
Clone 3b.ab1
Clone 2b.ab1
Clone 28b.ab1
Clone 2a.ab1
Clone 3a.ab1
Clone 28a.ab1

                   890       900       910       920       930       940       950       960       970        980
pOCT4-eGFP-2A-TCCCCCTGTCTCCGTCACCACTCTGGGCTCTCCCATGCATTCAAACGCTAGCATGGTGAGCAAGGGCGAGGAGCTGTTCACCGGGG-TGGTGCCCATCCT
              ~~~~~~~~~~~~~~~~~~~~~~~~~~~~~~~~~~~~~~~~~~~~~~~~~~~~~~~~~~~~~~~~~~~~~~~~~~~~~~~~~~~~~~~~~~~~~~~~~~~~
Consensus:    TCCCCCTGTCTCCGTCACCACTCTGGGCTCTCCCATGCATTCAAACGCTAGCATGGTGAGCAAGGGCGAGGAGCTGTTCACCGGGG-TGGTGCCCATCCT
              ~~~~~~~~~~~~~~~~~~~~~~~~~~~~~~~~~~~~~~~~~~~~~~~~~~~~~~~~~~~~~~~~~~~~~~~~~~~~~~~~~~~~~~~~~~~~~~~~~~~~
Clone 2c.ab1  TCCCCCTGTCTCCGTCACCACTCTGGGCTCTCCCATGCATTCAAACGCTAGCATGGTGAGCAAGGGCGAGGAGCTGTTCACCGGGG-TGGTGCCCATCCT
Clone 3c.ab1  TCCCCCTGTCTCCGTCACCACTCTGGGCTCTCCCATGCATTCAAACGCTAGCATGGTGAGCAAGGGCGAGGAGCTGTTCACCGGGGGTGGTGCCCATCCT
Clone 28c.ab1 TCCCCCTGTCTCCGTCACCACTCTGGGCTCTCCCATGCATTCAAACGCTAGCATGGTGAGCAAGGGCGAGGAGCTGTTCACCGGGGGTGGTGCCCATCCT
Clone 28d.ab1 TCCCCCTGTCTCCGTCACCACTCTGGGCTCTCCCATGCATTCAAACGCTAGCATGGTGAGCAAGGGCGAGGAGCTGTTCACCGGGG-TGGTGCCCATCCT
Clone 2d.ab1  TCCCCCTGTCTCCGTCACCACTCTGGGCTCTCCCATGCATTCAAACGCTAGCATGGTGAGCAAGGGCGAGGAGCTGTTCACCGGGG-TGGTGCCCATCCT
Clone 3d.ab1  TCCCCCTGTCTCCGTCACCACTCTGGGCTCTCCCATGCATTCAAACGCTAGCATGGTGAGCAAGGGCGAGGAGCTGTTCACCGGGG-TGGTGCCCATCCT
Clone 3b.ab1
Clone 2b.ab1
Clone 28b.ab1
Clone 2a.ab1
Clone 3a.ab1
Clone 28a.ab1

                    990      1000      1010       1020       1030       1040      1050      1060       1070
pOCT4-eGFP-2A-GGTCGAGCTGGACGGCGACGTAAACGGCCACAA-GTT-CAGCGTGTCCGG-CGAGGGCGAGGGCGATGCCACCTACGGC-AAGCTGACCCTGAA-GTT-C
              ~~~~~~~~~~~~~~~~~~~~~~~~~~~~~~~~~~~~~~~~~~~~~~~~~~~~~~~~~~~~~~~~~~~~~~~~~~~~~~~~~~~~~~~~~~~~~~~~~~~~
Consensus:    GGTCGAGCTGGACGGCGACGTAAaCGGCCACAA-GTT-CAGCGTGTCCGG-CGAGGGCGAGGGCGATGCCACCTACGGC-AAGCTGACCCTGAA-GTT-C
              ~~~~~~~~~~~~~~~~~~~~~~~~~~~~~~~~~~~~~~~~~~~~~~~~~~~~~~~~~~~~~~~~~~~~~~~~~~~~~~~~~~~~~~~~~~~~~~~~~~~~
Clone 2c.ab1  GGTCGAGCTGGACGGCGACGTAACGGGCCACAA-GTTTCAGCGTGTCCGGGCGAGGGCGAGGGCGATGCCACCTACGGC-AAGCTGAACCTGGA-G-T-C
Clone 3c.ab1  GGTCGAGCTGGACGGCGACGTAA-CGGCCACAA-GTT-CAGCGTGTCCGG-CGAGGGCGAGGGCGATGCCACCTACGGC-AAGCTGACCCTGAAAGTTTC
Clone 28c.ab1 GGTCGAGCTGGACGGCGACGTAA-CGGCCACAAAGTT-CAGCGTGTCCGG-CGAGGGCGAGGGCGATGCCACCTACGGCCAAGCTGACCCTGAA-GTT-C
Clone 28d.ab1 GGTCGAGCTGGACGGCGACGTAAACGGCCACAA-GTT-CAGCGTGTCCGG-CGAGGGCGAGGGCGATGCCACCTACGGC-AAGCTGACCCTGAA-GTT-C
Clone 2d.ab1  GGTCGAGCTGGACGGCGACGTAAACGGCCACAA-GTT-CAGCGTGTCCGG-CGAGGGCGAGGGCGATGCCACCTACGGC-AAGCTGACCCTGAA-GTT-C
Clone 3d.ab1  GGTCGAGCTGGACGGCGACGTAAACGGCCACAA-GTT-CAGCGTGTCCGG-CGAGGGCGAGGGCGATGCCACCTACGGC-AAGCTGACCCTGAA-GTT-C
Clone 3b.ab1
Clone 2b.ab1
Clone 28b.ab1
Clone 2a.ab1
Clone 3a.ab1
Clone 28a.ab1

               1080      1090        1100        1110        1120       1130       1140      1150      1160
pOCT4-eGFP-2A-ATCTGCACCACC-GGCAA-GCTGCCC-GTGCCC-TGGCCCACCC-T-CGTGACC-ACCC-TGACCTACGGCGTGCAGTGCTTCAGCCGCTACCCCGACCA
              ~~~~~~~~~~~~~~~~~~~~~~~~~~~~~~~~~~~~~~~~~~~~~~~~~~~~~~~~~~~~~~~~~~~~~~~~~~~~~~~~~~~~~~~~~~~~~~~~~~~~
Consensus:    ATCTGCACCACC-GGCAA-GCTGCCC-GTGCCC-TGGCCCACCC-T-CGTGACC-ACCC-TGACCTACGGCGTGCAGTGCTTCAGCCGCTACCCCGACCA
              ~~~~~~~~~~~~~~~~~~~~~~~~~~~~~~~~~~~~~~~~~~~~~~~~~~~~~~~~~~~~~~~~~~~~~~~~~~~~~~~~~~~~~~~~~~~~~~~~~~~~
Clone 2c.ab1  ATCTGCA-CA-C-GGCCA-GCTGCCC-GTGCCC-TGGCCCACCCCTTCGTGAC
Clone 3c.ab1  ATCTGCACCACC-GGCAA-GCTGCCCCGTGCCCCTGGCCCACCCCT-CGTGACCCACCCCTG
Clone 28c.ab1 ATCTGCACCACCCGGCAAAGCTGCCC-GTGCCC-TGGCCCACCC-T-CGTGACa
Clone 28d.ab1 ATCTGCACCACC-GGCAA-GCTGCCC-GTGCCC-TGGCCCACCC-T-CGTGACC-ACCC-TGACCTACGGCGTGCAGTGCTTCAGCCGCTACCCCGACCA
Clone 2d.ab1  ATCTGCACCACC-GGCAA-GCTGCCC-GTGCCC-TGGCCCACCC-T-CGTGACC-ACCC-TGACCTACGGCGTGCAGTGCTTCAGCCGCTACCCCGACCA
Clone 3d.ab1  ATCTGCACCACC-GGCAA-GCTGCCC-GTGCCC-TGGCCCACCC-T-CGTGACC-ACCC-TGACCTACGGCGTGCAGTGCTTCAGCCGCTACCCCGACCA
Clone 3b.ab1
Clone 2b.ab1
Clone 28b.ab1
Clone 2a.ab1
Clone 3a.ab1
Clone 28a.ab1

             1170      1180      1190      1200      1210      1220      1230      1240      1250      1260
pOCT4-eGFP-2A-CATGAAGCAGCACGACTTCTTCAAGTCCGCCATGCCCGAAGGCTACGTCCAGGAGCGCACCATCTTCTTCAAGGACGACGGCAACTACAAGACCCGCGCC
```

```
                ~~~~~~~~~~~~~~~~~~~~~~~~~~~~~~~~~~~~~~~~~~~~~~~~~~~~~~~~~~~~~~~~~~~~~~~~~~~~~~~~~~~~~~~~~~~~~~~~
Consensus:      CATGAAGCAGCACGACTTCTTCAAGTCCGCCATGCCCGAAGGCTAC-TCCAG
                ~~~~~~~~~~~~~~~~~~~~~~~~~~~~~~~~~~~~~~~~~~~~~~~~~~~~~~~~~~~~~~~~~~~~~~~~~~~~~~~~~~~~~~~~~~~~~~~~
Clone 2c.ab1
Clone 3c.ab1
Clone 28c.ab1
Clone 28d.ab1 CATGAAGCAGCACGACTTCTTCAAGTCCGCCATGCCCGAAGGCTACtctctctacgtttcctg
Clone 2d.ab1  CATGAAGCAGCACGACTTCTTCAAGTCCGCCATGCCCGAAGGCTAC-TCCAGattccgt
Clone 3d.ab1  CATGAAGCAGCACGACTTCTTCAAGTCCGCCATGCCCGAAGGCTACtctcttggatttcttt
Clone 3b.ab1
Clone 2b.ab1
Clone 28b.ab1
Clone 2a.ab1
Clone 3a.ab1
Clone 28a.ab1

           1270      1280      1290      1300      1310      1320      1330      1340      1350      1360
pOCT4-eGFP-2A-GAGGTGAAGTTCGAGGGCGACACCCTGGTGAACCGCATCGAGCTGAAGGGCATCGACTTCAAGGAGGACGGCAACATCCTGGGGCACAAGCTGGAGTACA
              ~~~~~~~~~~~~~~~~~~~~~~~~~~~~~~~~~~~~~~~~~~~~~~~~~~~~~~~~~~~~~~~~~~~~~~~~~~~~~~~~~~~~~~~~~~~~~~~~
Consensus:
              ~~~~~~~~~~~~~~~~~~~~~~~~~~~~~~~~~~~~~~~~~~~~~~~~~~~~~~~~~~~~~~~~~~~~~~~~~~~~~~~~~~~~~~~~~~~~~~~~
Clone 2c.ab1
Clone 3c.ab1
Clone 28c.ab1
Clone 28d.ab1
Clone 2d.ab1
Clone 3d.ab1
Clone 3b.ab1
Clone 2b.ab1
Clone 28b.ab1
Clone 2a.ab1
Clone 3a.ab1
Clone 28a.ab1

           1370      1380      1390      1400      1410      1420      1430      1440      1450      1460
pOCT4-eGFP-2A-ACTACAACAGCCACAACGTCTATATCATGGCCGACAAGCAGAAGAACGGCATCAAGGTGAACTTCAAGATCCGCCACAACATCGAGGACGGCAGCGTGCA
              ~~~~~~~~~~~~~~~~~~~~~~~~~~~~~~~~~~~~~~~~~~~~~~~~~~~~~~~~~~~~~~~~~~~~~~~~~~~~~~~~~~~~~~~~~~~~~~~~
Consensus:
              ~~~~~~~~~~~~~~~~~~~~~~~~~~~~~~~~~~~~~~~~~~~~~~~~~~~~~~~~~~~~~~~~~~~~~~~~~~~~~~~~~~~~~~~~~~~~~~~~
Clone 2c.ab1
Clone 3c.ab1
Clone 28c.ab1
Clone 28d.ab1
Clone 2d.ab1
Clone 3d.ab1
Clone 3b.ab1
Clone 2b.ab1
Clone 28b.ab1
Clone 2a.ab1
Clone 3a.ab1
Clone 28a.ab1

           1470      1480      1490      1500      1510      1520      1530      1540      1550      1560
pOCT4-eGFP-2A-GCTCGCCGACCACTACCAGCAGAACACCCCCATCGGCGACGGCCCCGTGCTGCTGCCCGACAACCACTACCTGAGCACCCAGTCCGCCCTGAGCAAAGAC
              ~~~~~~~~~~~~~~~~~~~~~~~~~~~~~~~~~~~~~~~~~~~~~~~~~~~~~~~~~~~~~~~~~~~~~~~~~~~~~~~~~~~~~~~~~~~~~~~~
Consensus:
              ~~~~~~~~~~~~~~~~~~~~~~~~~~~~~~~~~~~~~~~~~~~~~~~~~~~~~~~~~~~~~~~~~~~~~~~~~~~~~~~~~~~~~~~~~~~~~~~~
Clone 2c.ab1
Clone 3c.ab1
Clone 28c.ab1
Clone 28d.ab1
Clone 2d.ab1
Clone 3d.ab1
Clone 3b.ab1
Clone 2b.ab1
Clone 28b.ab1
Clone 2a.ab1
Clone 3a.ab1
Clone 28a.ab1

           1570      1580      1590      1600      1610      1620      1630      1640      1650      1660
pOCT4-eGFP-2A-CCCAACGAGAAGCGCGATCACATGGTCCTGCTGGAGTTCGTGACCGCCGCCGGGATCACTCTCGGCATGGACGAGCTGTACAAGTACAGCGGTCGCGACT
              ~~~~~~~~~~~~~~~~~~~~~~~~~~~~~~~~~~~~~~~~~~~~~~~~~~~~~~~~~~~~~~~~~~~~~~~~~~~~~~~~~~~~~~~~~~~~~~~~
Consensus:
              ~~~~~~~~~~~~~~~~~~~~~~~~~~~~~~~~~~~~~~~~~~~~~~~~~~~~~~~~~~~~~~~~~~~~~~~~~~~~~~~~~~~~~~~~~~~~~~~~
Clone 2c.ab1
Clone 3c.ab1
Clone 28c.ab1
Clone 28d.ab1
Clone 2d.ab1
Clone 3d.ab1
Clone 3b.ab1
Clone 2b.ab1
Clone 28b.ab1
Clone 2a.ab1
Clone 3a.ab1
Clone 28a.ab1

           1670      1680      1690      1700      1710      1720      1730      1740      1750      1760
pOCT4-eGFP-2A-CTAGAGTCGACGGTTCTGGTAAGCAAACTTTGAATTTTGACCTTCTTAAGCTTGCGGGAGACGTCGAGTCCAACCCCGGGCCCGCATGCAAGCTTCAGCT
              ~~~~~~~~~~~~~~~~~~~~~~~~~~~~~~~~~~~~~~~~~~~~~~~~~~~~~~~~~~~~~~~~~~~~~~~~~~~~~~~~~~~~~~~~~~~~~~~~
Consensus:
              ~~~~~~~~~~~~~~~~~~~~~~~~~~~~~~~~~~~~~~~~~~~~~~~~~~~~~~~~~~~~~~~~~~~~~~~~~~~~~~~~~~~~~~~~~~~~~~~~
Clone 2c.ab1
Clone 3c.ab1
Clone 28c.ab1
Clone 28d.ab1
Clone 2d.ab1
Clone 3d.ab1
Clone 3b.ab1
Clone 2b.ab1
Clone 28b.ab1
Clone 2a.ab1
Clone 3a.ab1
Clone 28a.ab1

           1770      1780      1790      1800      1810      1820      1830      1840      1850      1860
pOCT4-eGFP-2A-GAAGCTTACCATGACCGAGTACAAGCCCACGGTGCGCCTCGCCACCCGCGACGACGTCCCCAGGGCCGTACGCACCCTCGCCGCCGCGTTCGCCGACTAC
              ~~~~~~~~~~~~~~~~~~~~~~~~~~~~~~~~~~~~~~~~~~~~~~~~~~~~~~~~~~~~~~~~~~~~~~~~~~~~~~~~~~~~~~~~~~~~~~~~
```

```
Consensus:
              ~~~~~~~~~~~~~~~~~~~~~~~~~~~~~~~~~~~~~~~~~~~~~~~~~~~~~~~~~~~~~~~~~~~~~~~~~~~~~~~~~~~~~~~~~~~~~~~~~~~~
Clone 2c.ab1
Clone 3c.ab1
Clone 28c.ab1
Clone 28d.ab1
Clone 2d.ab1
Clone 3d.ab1
Clone 3b.ab1
Clone 2b.ab1
Clone 28b.ab1
Clone 2a.ab1
Clone 3a.ab1
Clone 28a.ab1

           1870      1880      1890      1900      1910      1920      1930      1940      1950      1960
pOCT4-eGFP-2A-CCCGCCACGCGCCACACCGTCGATCCGGACCGCCACATCGAGCGGGTCACCGAGCTGCAAGAACTCTTCCTCACGCGCGTCGGGCTCGACATCGGCAAGG
              ~~~~~~~~~~~~~~~~~~~~~~~~~~~~~~~~~~~~~~~~~~~~~~~~~~~~~~~~~~~~~~~~~~~~~~~~~~~~~~~~~~~~~~~~~~~~~~~~~~~~
Consensus:
              ~~~~~~~~~~~~~~~~~~~~~~~~~~~~~~~~~~~~~~~~~~~~~~~~~~~~~~~~~~~~~~~~~~~~~~~~~~~~~~~~~~~~~~~~~~~~~~~~~~~~
Clone 2c.ab1
Clone 3c.ab1
Clone 28c.ab1
Clone 28d.ab1
Clone 2d.ab1
Clone 3d.ab1
Clone 3b.ab1
Clone 2b.ab1
Clone 28b.ab1
Clone 2a.ab1
Clone 3a.ab1
Clone 28a.ab1

           1970      1980      1990      2000      2010      2020      2030      2040      2050      2060
pOCT4-eGFP-2A-TGTGGGTCGCGGACGACGGCGCCGCGGTGGCGGTCTGGACCACGCCGGAGAGCGTCGAAGCGGGGGCGGTGTTCGCCGAGATCGGCCCGCGCATGGCCGA
              ~~~~~~~~~~~~~~~~~~~~~~~~~~~~~~~~~~~~~~~~~~~~~~~~~~~~~~~~~~~~~~~~~~~~~~~~~~~~~~~~~~~~~~~~~~~~~~~~~~~~
Consensus:
              ~~~~~~~~~~~~~~~~~~~~~~~~~~~~~~~~~~~~~~~~~~~~~~~~~~~~~~~~~~~~~~~~~~~~~~~~~~~~~~~~~~~~~~~~~~~~~~~~~~~~
Clone 2c.ab1
Clone 3c.ab1
Clone 28c.ab1
Clone 28d.ab1
Clone 2d.ab1
Clone 3d.ab1
Clone 3b.ab1
Clone 2b.ab1
Clone 28b.ab1
Clone 2a.ab1
Clone 3a.ab1
Clone 28a.ab1

           2070      2080      2090      2100      2110      2120      2130      2140      2150      2160
pOCT4-eGFP-2A-GTTGAGCGGTTCCCGGCTGGCCGCGCAGCAACAGATGGAAGGCCTCCTGGCGCCGCACCGGCCCAAGGAGCCCGCGTGGTTCCTGGCCACCGTCGGCGTC
              ~~~~~~~~~~~~~~~~~~~~~~~~~~~~~~~~~~~~~~~~~~~~~~~~~~~~~~~~~~~~~~~~~~~~~~~~~~~~~~~~~~~~~~~~~~~~~~~~~~~~
Consensus:
              ~~~~~~~~~~~~~~~~~~~~~~~~~~~~~~~~~~~~~~~~~~~~~~~~~~~~~~~~~~~~~~~~~~~~~~~~~~~~~~~~~~~~~~~~~~~~~~~~~~~~
Clone 2c.ab1
Clone 3c.ab1
Clone 28c.ab1
Clone 28d.ab1
Clone 2d.ab1
Clone 3d.ab1
Clone 3b.ab1
Clone 2b.ab1
Clone 28b.ab1
Clone 2a.ab1
Clone 3a.ab1
Clone 28a.ab1

           2170      2180      2190      2200      2210      2220      2230      2240      2250      2260
pOCT4-eGFP-2A-TCGCCCGACCACCAGGGCAAGGGTCTGGGCAGCGCCGTCGTGCTCCCCGGAGTGGAGGCGGCCGAGCGCGCCGGGGTGCCCGCCTTCCTGGAGACCTCCG
              ~~~~~~~~~~~~~~~~~~~~~~~~~~~~~~~~~~~~~~~~~~~~~~~~~~~~~~~~~~~~~~~~~~~~~~~~~~~~~~~~~~~~~~~~~~~~~~~~~~~~
Consensus:
              ~~~~~~~~~~~~~~~~~~~~~~~~~~~~~~~~~~~~~~~~~~~~~~~~~~~~~~~~~~~~~~~~~~~~~~~~~~~~~~~~~~~~~~~~~~~~~~~~~~~~
Clone 2c.ab1
Clone 3c.ab1
Clone 28c.ab1
Clone 28d.ab1
Clone 2d.ab1
Clone 3d.ab1
Clone 3b.ab1                                                 ctgcactagctgactggcattatctactgtcaacagctaaaccggggatttct
Clone 2b.ab1                                                                           ttctcacccactctgccgtttgtgtata
Clone 28b.ab1                                                                                    ttgttgtatgtagtagat
Clone 2a.ab1
Clone 3a.ab1
Clone 28a.ab1

           2270      2280      2290       2300       2310       2320       2330       2340      2350      2360
pOCT4-eGFP-2A-CGCCCCGCAACCTCCCCTTCTA-CGAGCGGCTCGGCTT-CACCGTCACCGC-C-GACGTCGAGG-TGCCCGAAGGACCGCGCACCTGGTGCATGACCCGC
              ~~~~~~~~~~~~~~~~~~~~~~~~~~~~~~~~~~~~~~~~~~~~~~~~~~~~~~~~~~~~~~~~~~~~~~~~~~~~~~~~~~~~~~~~~~~~~~~~~~~~
Consensus:            AAACTCCTCTYTTA-CGAGCGGCTCGGCTT-CACCGTCACCGC-C-GACGTCGAGG-TGCCCGAAGGACCGCGCACcTGGTGCATGACCCGC
              ~~~~~~~~~~~~~~~~~~~~~~~~~~~~~~~~~~~~~~~~~~~~~~~~~~~~~~~~~~~~~~~~~~~~~~~~~~~~~~~~~~~~~~~~~~~~~~~~
Clone 2c.ab1
Clone 3c.ab1
Clone 28c.ab1
Clone 28d.ab1
Clone 2d.ab1
Clone 3d.ab1
Clone 3b.ab1  ggcaaatgttggcctttTTTTAGCTAGC-GCTC-GCTT-CA-CGTCA-CG--C-GACGTCGAGGGTGCCCGAAGGACCGCGCAC-TGGTGCATGACCCGC
Clone 2b.ab1  gtagataaAAACTCCTCTCTTA-CGAGCGGCTCGGCTT-CACCGTCACCGC-C-GACGTCGAGG-TGCCCGAAGGACCGCGCAC-TGGTGCATGACCCGC
Clone 28b.ab1 agtaatcaaaaaccctctctTA-CGAGCGGCTCGGCTTTCACCGTCACCGC-C-GACGTCGAGGGTGCCCGAAGGACCGCGCACGTGGTGCATGACCCGC
Clone 2a.ab1                                           tcctgatgCCGC-CGGACGTCGAGG-TGCCCGAAGGACCGCGCACCTGGTGCATGACCCGC
Clone 3a.ab1                                            gtctgtgaaag-A-GACGTCGAGG-TGCCCGAAGGACCGCGCACCTGGTGCATGACCCGC
Clone 28a.ab1                                               tctgaGCGC-GACGTCGAGG-TGCCCGAAGGACCGCGCACCTGGTGCATGACCCGC

                2370      2380      2390      2400      2410       2420       2430      2440       2450      2460
pOCT4-eGFP-2A-AAGCCCGGTGCCTGAGGCGCGCCGGTGCCTGCCCTTCTAGGAATGGGGG-ACAGGGGGAGGGG-AGGAGCTAGGGAAAGAAAA-CCTGGAGTTTGTGCCA
              ~~~~~~~~~~~~~~~~~~~~~~~~~~~~~~~~~~~~~~~~~~~~~~~~~~~~~~~~~~~~~~~~~~~~~~~~~~~~~~~~~~~~~~~~~~~~~~~~~~~~
Consensus:    AAGCCCGGTGCcTGAGGCGCGCCGGTGCCTGCCCTTCTAGGAATGGGGGgACAGGGGGAGGGG-AGGAGCTAGGGAAAGAAAA-CCTGGAGTTTGTGCCA
```

```
                ~~~~~~~~~~~~~~~~~~~~~~~~~~~~~~~~~~~~~~~~~~~~~~~~~~~~~~~~~~~~~~~~~~~~~~~~~~~~~~~~~~~~~~~~~~~~~~~~
Clone 2c.ab1
Clone 3c.ab1
Clone 28c.ab1
Clone 28d.ab1
Clone 2d.ab1
Clone 3d.ab1
Clone 3b.ab1    AAGCCCGGTGC-TGAGGCGCGCCGGTGCCTGCCCTTCTAGGAATGGGGGGACAGGGGGAGGGG-AGGAGCTAGGGAAAGAAAA-CCTGGAGTTTGTGCCA
Clone 2b.ab1    AAGCCCGGTGC-TGAGGCGCGCCGGTGCCTGCCCTTCTAGGAATGGGGGGACAGGGGGAGGGG-AGGAGCTAGGGAAAGAAAAACCTGGAGTTTGTGCCA
Clone 28b.ab1   AAGCCCGGTGC-TGAGGCGCGCCGGTGCCTGCCCTTCTAGGAATGGGGGGACAGGGGGAGGGGGAGGAGCTAGGGAAAGAAAA-CCTGGAGTTTGTGCCA
Clone 2a.ab1    AAGCCCGGTGCCTGAGGCGCGCCGGTGCCTGCCCTTCTAGGAATGGGGG-ACAGGGGGAGGGG-AGGAGCTAGGGAAAGAAAA-CCTGGAGTTTGTGCCA
Clone 3a.ab1    AAGCCCGGTGCCTGAGGCGCGCCGGTGCCTGCCCTTCTAGGAATGGGGG-ACAGGGGGAGGGG-AGGAGCTAGGGAAAGAAAA-CCTGGAGTTTGTGCCA
Clone 28a.ab1   AAGCCCGGTGCCTGAGGCGCGCCGGTGCCTGCCCTTCTAGGAATGGGGG-ACAGGGGGAGGGG-AGGAGCTAGGGAAAGAAAA-CCTGGAGTTTGTGCCA

                     2470      2480      2490      2500      2510      2520      2530      2540      2550      2560
pOCT4-eGFP-2A-  GGGTTTTTGGGATTAAGTTCTTCATTCACTAAGGAAGGAATTGGGAACACAAAGGGTGGGGGCAGGGGAGTTTGGGGCAACTGGTTGGAGGGAAGGTGAA
                ~~~~~~~~~~~~~~~~~~~~~~~~~~~~~~~~~~~~~~~~~~~~~~~~~~~~~~~~~~~~~~~~~~~~~~~~~~~~~~~~~~~~~~~~~~~~~~~~
Consensus:      GGGTTTTTGGGATTAAGTTCTTCATTCACTAAGGAAGGAATTGGGAACACAAAGGGTGGGGGCAGGGGAGTTTGGGGCAACTGGTTGGAGGGAAGGTGAA
                ~~~~~~~~~~~~~~~~~~~~~~~~~~~~~~~~~~~~~~~~~~~~~~~~~~~~~~~~~~~~~~~~~~~~~~~~~~~~~~~~~~~~~~~~~~~~~~~~
Clone 2c.ab1
Clone 3c.ab1
Clone 28c.ab1
Clone 28d.ab1
Clone 2d.ab1
Clone 3d.ab1
Clone 3b.ab1    GGGTTTTTGGGATTAAGTTCTTCATTCACTAAGGAAGGAATTGGGAACACAAAGGGTGGGGGCAGGGGAGTTTGGGGCAACTGGTTGGAGGGAAGGTGAA
Clone 2b.ab1    GGGTTTTTGGGATTAAGTTCTTCATTCACTAAGGAAGGAATTGGGAACACAAAGGGTGGGGGCAGGGGAGTTTGGGGCAACTGGTTGGAGGGAAGGTGAA
Clone 28b.ab1   GGGTTTTTGGGATTAAGTTCTTCATTCACTAAGGAAGGAATTGGGAACACAAAGGGTGGGGGCAGGGGAGTTTGGGGCAACTGGTTGGAGGGAAGGTGAA
Clone 2a.ab1    GGGTTTTTGGGATTAAGTTCTTCATTCACTAAGGAAGGAATTGGGAACACAAAGGGTGGGGGCAGGGGAGTTTGGGGCAACTGGTTGGAGGGAAGGTGAA
Clone 3a.ab1    GGGTTTTTGGGATTAAGTTCTTCATTCACTAAGGAAGGAATTGGGAACACAAAGGGTGGGGGCAGGGGAGTTTGGGGCAACTGGTTGGAGGGAAGGTGAA
Clone 28a.ab1   GGGTTTTTGGGATTAAGTTCTTCATTCACTAAGGAAGGAATTGGGAACACAAAGGGTGGGGGCAGGGGAGTTTGGGGCAACTGGTTGGAGGGAAGGTGAA

                     2570      2580      2590      2600      2610      2620      2630      2640      2650      2660
pOCT4-eGFP-2A-  GTTCAATGATGCTCTTGATTTTAATCCCACATCATGTATCACTTTTTTCTTAAATAAAGAAGCCTGGGACACAGTAGATAGACACACTTATCTTGGTTTG
                ~~~~~~~~~~~~~~~~~~~~~~~~~~~~~~~~~~~~~~~~~~~~~~~~~~~~~~~~~~~~~~~~~~~~~~~~~~~~~~~~~~~~~~~~~~~~~~~~
Consensus:      GTTCAATGATGCTCTTGATTTTAATCCCACATCATGTATCACTTTTTTCTTAAATAAAGAAGCTTGGGACACAGTAGATAGACACACTTATCTTGGTTTG
                ~~~~~~~~~~~~~~~~~~~~~~~~~~~~~~~~~~~~~~~~~~~~~~~~~~~~~~~~~~~~~~~~~~~~~~~~~~~~~~~~~~~~~~~~~~~~~~~~
Clone 2c.ab1
Clone 3c.ab1
Clone 28c.ab1
Clone 28d.ab1
Clone 2d.ab1
Clone 3d.ab1
Clone 3b.ab1    GTTCAATGATGCTCTTGATTTTAATCCCACATCATGTATCACTTTTTTCTTAAATAAAGAAGCTTGGGACACAGTAGATAGACACACTTATCTTGGTTTG
Clone 2b.ab1    GTTCAATGATGCTCTTGATTTTAATCCCACATCATGTATCACTTTTTTCTTAAATAAAGAAGCCTGGGACACAGTAGATAGACACACTTATCTTGGTTTG
Clone 28b.ab1   GTTCAATGATGCTCTTGATTTTAATCCCACATCATGTATCACTTTTTTCTTAAATAAAGAAGCTTGGGACACAGTAGATAGACACACTTATCTTGGTTTG
Clone 2a.ab1    GTTCAATGATGCTCTTGATTTTAATCCCACATCATGTATCACTTTTTTCTTAAATAAAGAAGCCTGGGACACAGTAGATAGACACACTTATCTTGGTTTG
Clone 3a.ab1    GTTCAATGATGCTCTTGATTTTAATCCCACATCATGTATCACTTTTTTCTTAAATAAAGAAGCTTGGGACACAGTAGATAGACACACTTATCTTGGTTTG
Clone 28a.ab1   GTTCAATGATGCTCTTGATTTTAATCCCACATCATGTATCACTTTTTTCTTAAATAAAGAAGCTTGGGACACAGTAGATAGACACACTTATCTTGGTTTG

                     2670      2680      2690      2700      2710      2720      2730      2740      2750      2760
pOCT4-eGFP-2A-  TCCTTCAGTTACTGAGGTAGGGATGGGAATATCCAATGCTCATACCCAAGTGACCCTGAAACTAAGGTGCCATTTACACTCCTTAAGGTCACACAACATC
                ~~~~~~~~~~~~~~~~~~~~~~~~~~~~~~~~~~~~~~~~~~~~~~~~~~~~~~~~~~~~~~~~~~~~~~~~~~~~~~~~~~~~~~~~~~~~~~~~
Consensus:      TCCTTCAGTTACTGAGGTGGGGATGGGAATATCCAATGCTCATACCCAAGTGACCCTGAAACTAAGGTGCCATTTACACTCCTTAAGGTCACACAACATC
                ~~~~~~~~~~~~~~~~~~~~~~~~~~~~~~~~~~~~~~~~~~~~~~~~~~~~~~~~~~~~~~~~~~~~~~~~~~~~~~~~~~~~~~~~~~~~~~~~
Clone 2c.ab1
Clone 3c.ab1
Clone 28c.ab1
Clone 28d.ab1
Clone 2d.ab1
Clone 3d.ab1
Clone 3b.ab1    TCCTTCAGTTACTGAGGTGGGGATGGGAATATCCAATGCTCATACCCAAGTGACCCTGAAACTAAGGTGCCATTTACACTCCTTAAGGTCACACAACATC
Clone 2b.ab1    TCCTTCAGTTACTGAGGTGGGGATGGGAATATCCAATGCTCATACCCAAGTGACCCTGAAACTAAGGTGCCATTTACACTCCTTAAGGTCACACAACATC
Clone 28b.ab1   TCCTTCAGTTACTGAGGTGGGGATGGGAATATCCAATGCTCATACCCAAGTGACCCTGAAACTAAGGTGCCATTTACACTCCTTAAGGTCACACAACATC
Clone 2a.ab1    TCCTTCAGTTACTGAGGTGGGGATGGGAATATCCAATGCTCATACCCAAGTGACCCTGAAACTAAGGTGCCATTTACACTCCTTAAGGTCACACAACATC
Clone 3a.ab1    TCCTTCAGTTACTGAGGTGGGGATGGGAATATCCAATGCTCATACCCAAGTGACCCTGAAACTAAGGTGCCATTTACACTCCTTAAGGTCACACAACATC
Clone 28a.ab1   TCCTTCAGTTACTGAGGTGGGGATGGGAATATCCAATGCTCATACCCAAGTGACCCTGAAACTAAGGTGCCATTTACACTCCTTAAGGTCACACAACATC

                     2770      2780      2790      2800      2810      2820      2830      2840      2850      2860
pOCT4-eGFP-2A-  AGAGGGAGAGCTGGGATTGCAGCCAAGTTTATTTGTACAGGGCCCTGTGATAGGCTAGTTCCCAAAAGCCTGTGATGCAAGAACTTTTGCCCATAGACTC
                ~~~~~~~~~~~~~~~~~~~~~~~~~~~~~~~~~~~~~~~~~~~~~~~~~~~~~~~~~~~~~~~~~~~~~~~~~~~~~~~~~~~~~~~~~~~~~~~~
Consensus:      AGAGGGAGAGCTGGGATTGCAGCCAAGTTTATTTGTACAGGGCCCTGTGATAGGCTAGTTCCCAAAAGCCTGTGATGCAAGAACTTTTGCCCATAGACTC
                ~~~~~~~~~~~~~~~~~~~~~~~~~~~~~~~~~~~~~~~~~~~~~~~~~~~~~~~~~~~~~~~~~~~~~~~~~~~~~~~~~~~~~~~~~~~~~~~~
Clone 2c.ab1
Clone 3c.ab1
Clone 28c.ab1
Clone 28d.ab1
Clone 2d.ab1
Clone 3d.ab1
Clone 3b.ab1    AGAGGGAGAGCTGGGATTGCAGCCAAGTTTATTTGTACAGGGCCCTGTGATAGGCTAGTTCCCAAAAGCCTGTGATGCAAGAACTTTTGCCCATAGACTC
Clone 2b.ab1    AGAGGGAGAGCTGGGATTGCAGCCAAGTTTATTTGTACAGGGCCCTGTGATAGGCTAGTTCCCAAAAGCCTGTGATGCAAGAACTTTTGCCCATAGACTC
Clone 28b.ab1   AGAGGGAGAGCTGGGATTGCAGCCAAGTTTATTTGTACAGGGCCCTGTGATAGGCTAGTTCCCAAAAGCCTGTGATGCAAGAACTTTTGCCCATAGACTC
Clone 2a.ab1    AGAGGGAGAGCTGGGATTGCAGCCAAGTTTATTTGTACAGGGCCCTGTGATAGGCTAGTTCCCAAAAGCCTGTGATGCAAGAACTTTTGCCCATAGACTC
Clone 3a.ab1    AGAGGGAGAGCTGGGATTGCAGCCAAGTTTATTTGTACAGGGCCCTGTGATAGGCTAGTTCCCAAAAGCCTGTGATGCAAGAACTTTTGCCCATAGACTC
Clone 28a.ab1   AGAGGGAGAGCTGGGATTGCAGCCAAGTTTATTTGTACAGGGCCCTGTGATAGGCTAGTTCCCAAAAGCCTGTGATGCAAGAACTTTTGCCCATAGACTC

                     2870      2880      2890      2900      2910      2920      2930      2940      2950      2960
pOCT4-eGFP-2A-  AGTCACCATGTAGCTGTTACCTGTTCAGAGCTGGCTTTTTGCTTTCCCACCCTACTCTGGAATTCTTAAATGGCTTTATACTTAGAAATCATCTTATTTC
                ~~~~~~~~~~~~~~~~~~~~~~~~~~~~~~~~~~~~~~~~~~~~~~~~~~~~~~~~~~~~~~~~~~~~~~~~~~~~~~~~~~~~~~~~~~~~~~~~
Consensus:      AGTCACCATGTAGCTGTTACCTGTTCAGAGCTGGCTTTTTGCTTTCCCACCCTACTCTGGAATTCTTAAATGGCTTTATACTTAGAAATCATCTTATTTC
                ~~~~~~~~~~~~~~~~~~~~~~~~~~~~~~~~~~~~~~~~~~~~~~~~~~~~~~~~~~~~~~~~~~~~~~~~~~~~~~~~~~~~~~~~~~~~~~~~
Clone 2c.ab1
Clone 3c.ab1
Clone 28c.ab1
Clone 28d.ab1
Clone 2d.ab1
Clone 3d.ab1
Clone 3b.ab1    AGTCACCATGTAGCTGTTACCTGTTCAGAGCTGGCTTTTTGCTTTCCCACCCTACTCTGGAATTCTTAAATGGCTTTATACTTAGAAATCATCTTATTTC
Clone 2b.ab1    AGTCACCATGTAGCTGTTACCTGTTCAGAGCTGGCTTTTTGCTTTCCCACCCTACTCTGGAATTCTTAAATGGCTTTATACTTAGAAATCATCTTATTTC
Clone 28b.ab1   AGTCACCATGTAGCTGTTACCTGTTCAGAGCTGGCTTTTTGCTTTCCCACCCTACTCTGGAATTCTTAAATGGCTTTATACTTAGAAATCATCTTATTTC
Clone 2a.ab1    AGTCACCATGTAGCTGTTACCTGTTCAGAGCTGGCTTTTTGCTTTCCCACCCTACTCTGGAATTCTTAAATGGCTTTATACTTAGAAATCATCTTATTTC
Clone 3a.ab1    AGTCACCATGTAGCTGTTACCTGTTCAGAGCTGGCTTTTTGCTTTCCCACCCTACTCTGGAATTCTTAAATGGCTTTATACTTAGAAATCATCTTATTTC
Clone 28a.ab1   AGTCACCATGTAGCTGTTACCTGTTCAGAGCTGGCTTTTTGCTTTCCCACCCTACTCTGGAATTCTTAAATGGCTTTATACTTAGAAATCATCTTATTTC

                     2970      2980      2990      3000      3010      3020      3030      3040      3050      3060
pOCT4-eGFP-2A-  TGTTGAACCTAGATCACCCCAACCAGAAACTTCTATTAATACTTTGTGCTTTCTTGATACCAGGGTCTATTTGGTTTCCACTTAAGGTTTTTGCATACTC
                ~~~~~~~~~~~~~~~~~~~~~~~~~~~~~~~~~~~~~~~~~~~~~~~~~~~~~~~~~~~~~~~~~~~~~~~~~~~~~~~~~~~~~~~~~~~~~~~~
Consensus:      TGTTGAACCTAGATCACCCCAACCAGAAACTTCTATTAATACTTTGTGCTTTCTTGATACCAGGGTCTATTTGGTTTCCACTTAAGGTTTTTGCATACTC
                ~~~~~~~~~~~~~~~~~~~~~~~~~~~~~~~~~~~~~~~~~~~~~~~~~~~~~~~~~~~~~~~~~~~~~~~~~~~~~~~~~~~~~~~~~~~~~~~~
```

```
Clone 2c.ab1
Clone 3c.ab1
Clone 28c.ab1
Clone 28d.ab1
Clone 2d.ab1
Clone 3d.ab1
Clone 3b.ab1  TGTTGAACCTAGATCACCCCAACCAGAAACTTCTATTAATACTTTGTGCTTTCTTGATACCAGGGTCTATTTGGTTTCCACTTAAGGTTTTTGCATACTC
Clone 2b.ab1  TGTTGAACCTAGATCACCCCAACCAGAAACTTCTATTAATACTTTGTGCTTTCTTGATACCAGGGTCTATTTGGTTTCCACTTAAGGTTTTTGCATACTC
Clone 28b.ab1 TGTTGAACCTAGATCACCCCAACCAGAAACTTCTATTAATACTTTGTGCTTTCTTGATACCAGGGTCTATTTGGTTTCCACTTAAGGTTTTTGCATACTC
Clone 2a.ab1  TGTTGAACCTAGATCACCCCAACCAGAAACTTCTATTAATACTTTGTGCTTTCTTGATACCAGGGTCTATTTGGTTTCCACTTAAGGTTTTTGCATACTC
Clone 3a.ab1  TGTTGAACCTAGATCACCCCAACCAGAAACTTCTATTAATACTTTGTGCTTTCTTGATACCAGGGTCTATTTGGTTTCCACTTAAGGTTTTTGCATACTC
Clone 28a.ab1 TGTTGAACCTAGATCACCCCAACCAGAAACTTCTATTAATACTTTGTGCTTTCTTGATACCAGGGTCTATTTGGTTTCCACTTAAGGTTTTTGCATACTC

                    3070      3080      3090      3100      3110      3120      3130      3140      3150      3160
pOCT4-eGFP-2A-TGCCCATAAGTGACTCATTAGTTACTCAAGTTTTATTCCTGGCTCTGCCACTAGTTCATTAGGGGTCTTTGCCCCAGAGTCATTTCTTCCATTTTAAAAA
              ~~~~~~~~~~~~~~~~~~~~~~~~~~~~~~~~~~~~~~~~~~~~~~~~~~~~~~~~~~~~~~~~~~~~~~~~~~~~~~~~~~~~~~~~~~~~~~~~~~~~
Consensus:    TGCCCATAAGTGACTCATTAGTTACTCAAGTTTTATTCCTGGCTCTGCCACTAGTTCATTAGGGGTCTTTGCCCCAGAGTCATTTCTTCCATGTAAAAAA
              ~~~~~~~~~~~~~~~~~~~~~~~~~~~~~~~~~~~~~~~~~~~~~~~~~~~~~~~~~~~~~~~~~~~~~~~~~~~~~~~~~~~~~~~~~~~~~~~~~~~~
Clone 2c.ab1
Clone 3c.ab1
Clone 28c.ab1
Clone 28d.ab1
Clone 2d.ab1
Clone 3d.ab1
Clone 3b.ab1  TGCCCATAAGTGACTCATTAGTTACTCAAGTTTTATTCCTGGCTCTGCCACTAGTTCATTAGGGGTCTTTGCCCCAGAGTCATTTCTTCCATGTAAAAAA
Clone 2b.ab1  TGCCCATAAGTGACTCATTAGTTACTCAAGTTTTATTCCTGGCTCTGCCACTAGTTCATTAGGGGTCTTTGCCCCAGAGTCATTTCTTCCATGTAAAAAA
Clone 28b.ab1 TGCCCATAAGTGACTCATTAGTTACTCAAGTTTTATTCCTGGCTCTGCCACTAGTTCATTAGGGGTCTTTGCCCCAGAGTCATTTCTTCCATGTAAAAAA
Clone 2a.ab1  TGCCCATAAGTGACTCATTAGTTACTCAAGTTTTATTCCTGGCTCTGCCACTAGTTCATTAGGGGTCTTTGCCCCAGAGTCATTTCTTCCATGTAAAAAA
Clone 3a.ab1  TGCCCATAAGTGACTCATTAGTTACTCAAGTTTTATTCCTGGCTCTGCCACTAGTTCATTAGGGGTCTTTGCCCCAGAGTCATTTCTTCCATGTAAAAAA
Clone 28a.ab1 TGCCCATAAGTGACTCATTAGTTACTCAAGTTTTATTCCTGGCTCTGCCACTAGTTCATTAGGGGTCTTTGCCCCAGAGTCATTTCTTCCATGTAAAAAA

                     3170      3180      3190      3200      3210       3220      3230        3240      3250
pOCT4-eGFP-2A--CTTGGGCTCATTAAATCTAGGTAGGAAAGGGCGGGTGTGGCAGGTTTTAATAGAACA-GGTCAAGATAAGGC-TTT-ATTT-CTATAGAAATGATGCTT
              ~~~~~~~~~~~~~~~~~~~~~~~~~~~~~~~~~~~~~~~~~~~~~~~~~~~~~~~~~~~~~~~~~~~~~~~~~~~~~~~~~~~~~~~~~~~~~~~~~~~~
Consensus:    ACTTGGGCTCATTAAATCTAGGTAGGAAAGGGCGGATGTGGCAGGTTTTAATAGAACA-GGTCAAGATAAGGC-TTT-ATTTTCTATAGAAATGATGCTT
              ~~~~~~~~~~~~~~~~~~~~~~~~~~~~~~~~~~~~~~~~~~~~~~~~~~~~~~~~~~~~~~~~~~~~~~~~~~~~~~~~~~~~~~~~~~~~~~~~~~~~
Clone 2c.ab1
Clone 3c.ab1
Clone 28c.ab1
Clone 28d.ab1
Clone 2d.ab1
Clone 3d.ab1
Clone 3b.ab1  ACTTGGGCTCATTAAATCTAGGTAGGAAAGGGCGGATGTGGCAGGTTTTAATAGAACA-GGTCAAGATAAGGC-TTT-ATgccctacccgc
Clone 2b.ab1  ACTTGGGCTCATTAAATCTAGGTAGGAAAGGGCGGATGTGGCAGGTTTTAATAGAACA-GGTCAAGATAAGGCATTT-ATgccctacttaa
Clone 28b.ab1 ACTTGGGCTCATTAAATCTAGGTAGGAAAGGGCGGATGTGGCAGGTTTTAATAGAACA-GGTCtgcgacaagg-cct-gctg-catacccgaccttc
Clone 2a.ab1  ACTTGGGCTCATTAAATCTAGGTAGGAAAGGGCGGATGTGGCAGGTTTTAATAGAACA-GGTCAAGATAAGGC-TTTTATTTTCTATAGAAATGATGCTT
Clone 3a.ab1  ACTTGGGCTCATTAAATCTAGGTAGG-AAGGGCGGATGTGGCAGGTTTTAATAGAACACGGTCAAGATAA-GC-TTT-ATTTTCTATAGAAATGATGCTT
Clone 28a.ab1 -aaacttgggctcattaaatctaggtaggaaaggggcggatgtggcaggttttaatag-aacaggtcaagata-agg-cttt-atttctatagaaatgat

                3260      3270      3280      3290      3300      3310
pOCT4-eGFP-2A-T-GACA-ATAGTTTGGCTTGGTGTAAGGCTCACAAAAGAAAATCACATGTACCATGTGTGGGTTA
              ~~~~~~~~~~~~~~~~~~~~~~~~~~~~~~~~~~~~~~~~~~~~~~~~~~~~~~~~~~~~~~~~~~
Consensus:    TtGACATAT
              ~~~~~~~~~~~~~~~~~~~~~~~~~~~~~~~~~~~~~~~~~~~~~~~~~~~~~~~~~~~~~~~~~~
Clone 2c.ab1
Clone 3c.ab1
Clone 28c.ab1
Clone 28d.ab1
Clone 2d.ab1
Clone 3d.ab1
Clone 3b.ab1
Clone 2b.ab1
Clone 28b.ab1
Clone 2a.ab1  TTGACATATgctgccccccccccccgagtggcacgcacccccccgggtgcggcgtactacatt
Clone 3a.ab1  T-GACAtgtcatcccccccgcccccaaaaaagtacatatcggacggcgtcgcgcttc
Clone 28a.ab1 g-cttt-gacatagtaaggcgcgtggcagcgccgttctcgaccgtcggcgaaacctatagagacc
```

Supplemental Figure S1b

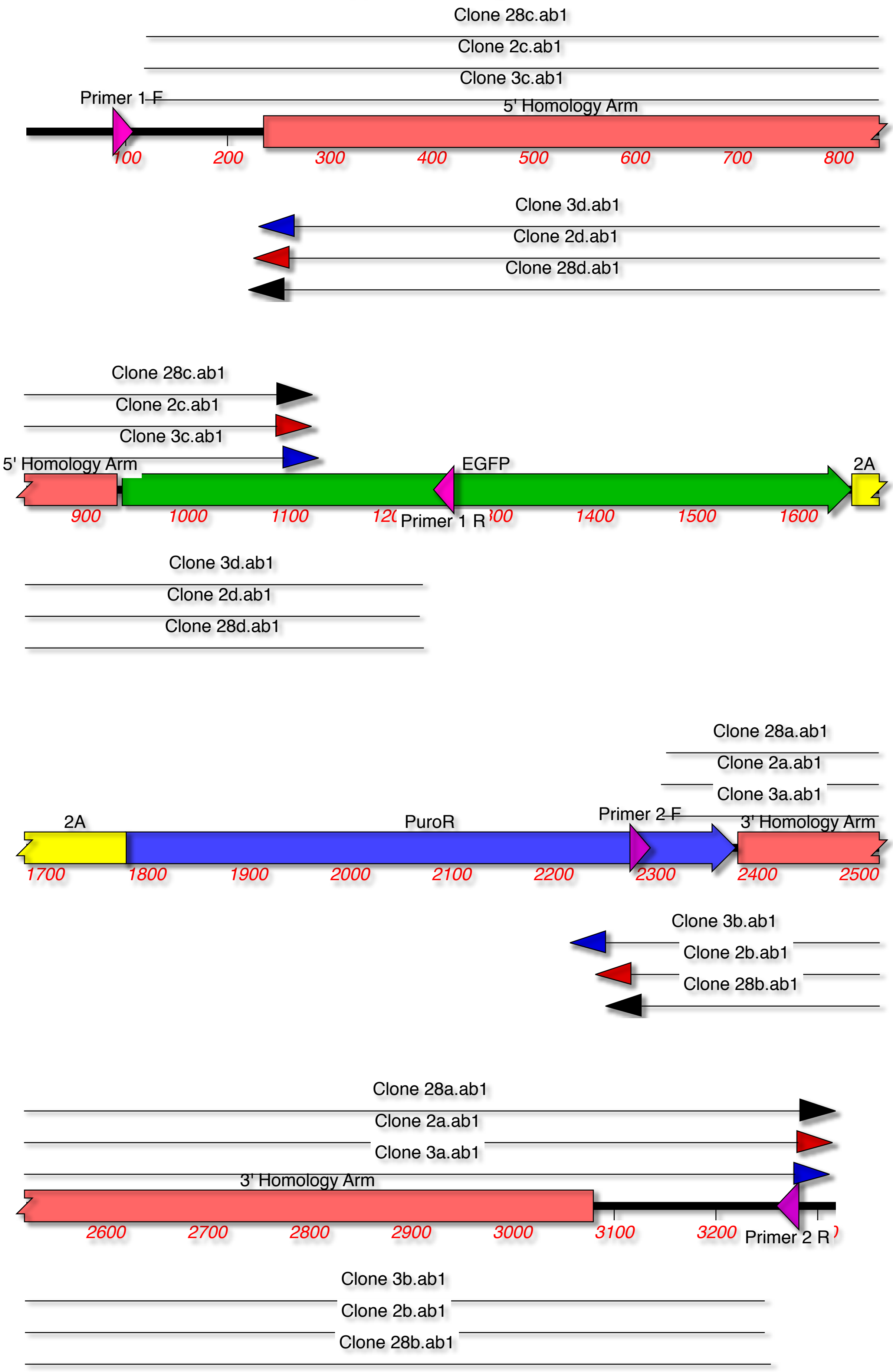

Supplement: Figure S1 — Sequencing analysis of OCT4-2, OCT4-3, and OCT4-28 hESC clones. Genomic DNA isolated from each cell line was sequenced using primers 5′F, 5′R, 3′F, and 3′R. (A) Aligned raw sequences from OCT4-2, OCT4-3, and OCT4-28 spanning homology arms. (B) Schematic of pOCT4-eGFP-2A-Puro genomic integration outlining the 5′ homology arm (pink box), eGFP (green box) and 3′ homology arm (pink box). Arrows highlight region of homology arm sequence alignment for OCT4-2 (red), OCT4-3 (blue) and OCT4-28 (black). Forward primers (hatched arrows) and reverse (solid arrows) genotyping and sequencing primers are highlighted. (PDF) [file pone.0114275.s001.pdf]
