# Supplementary figures and images for: TALEN/CRISPR-Mediated eGFP Knock-In Add-On at the OCT4 Locus Does Not Impact Differentiation of Human Embryonic Stem Cells towards Endoderm
Source: PLoS One. 2014 Dec 4;9(12):e114275. doi: 10.1371/journal.pone.0114275 (PMC4256397; doi:10.1371/journal.pone.0114275)

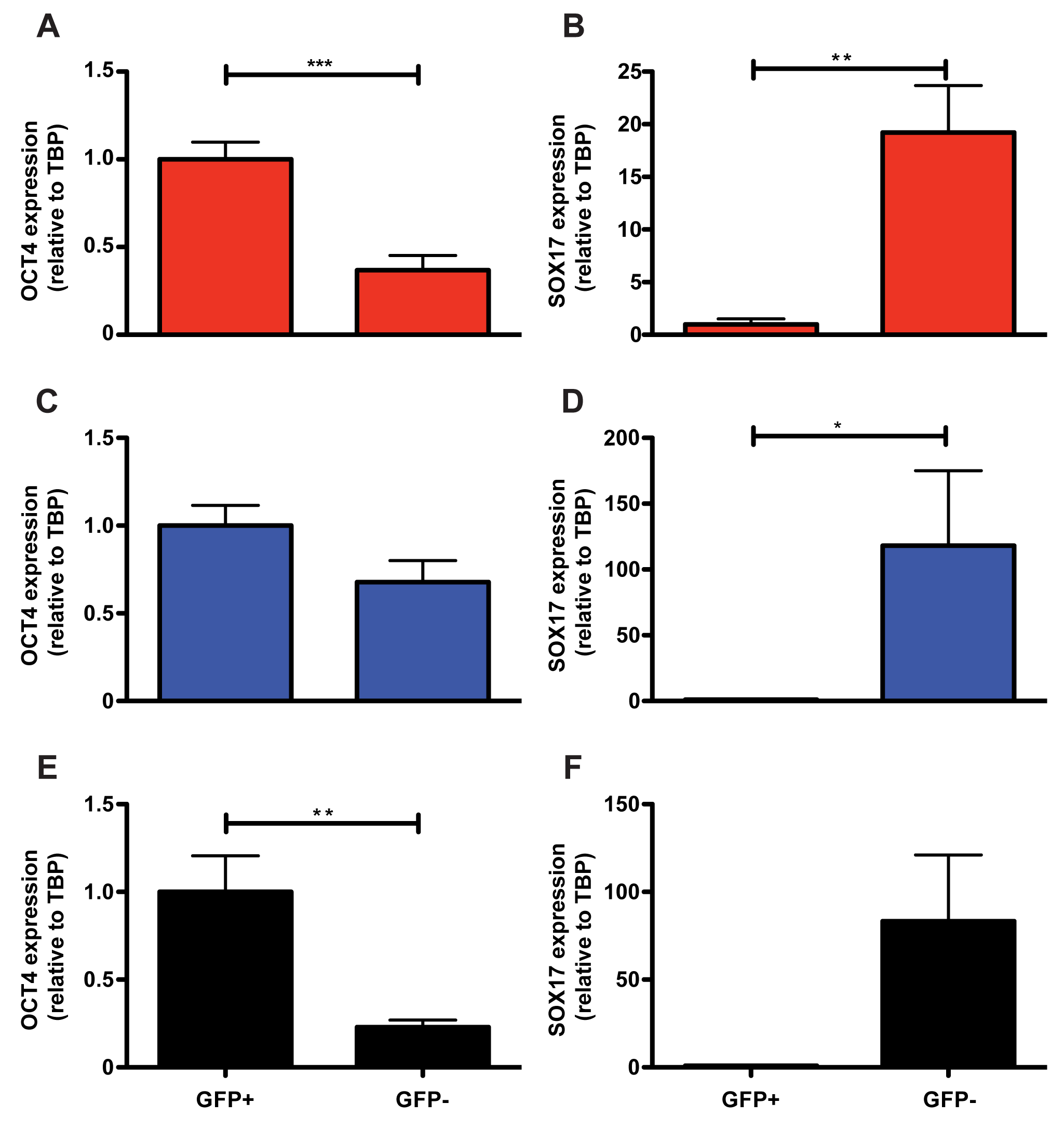

Supplement: Figure S2 — OCT4 and SOX17 expression in GFP+ and GFP− cells. Cells were trypsinized on the second day of differentiation to definitive endoderm and the GFP+ and GFP− populations were collected into TRIzol using the BD FACS Aria. RNA was isolated and cDNA synthesized before carrying out qPCR analysis for OCT4 and SOX17 using TBP as control gene. Statistical analysis was performed using a Student’s t-test. n≥3. *p<0.05, **p<0.01, ***p<0.001. (TIF) [file pone.0114275.s002.tif]

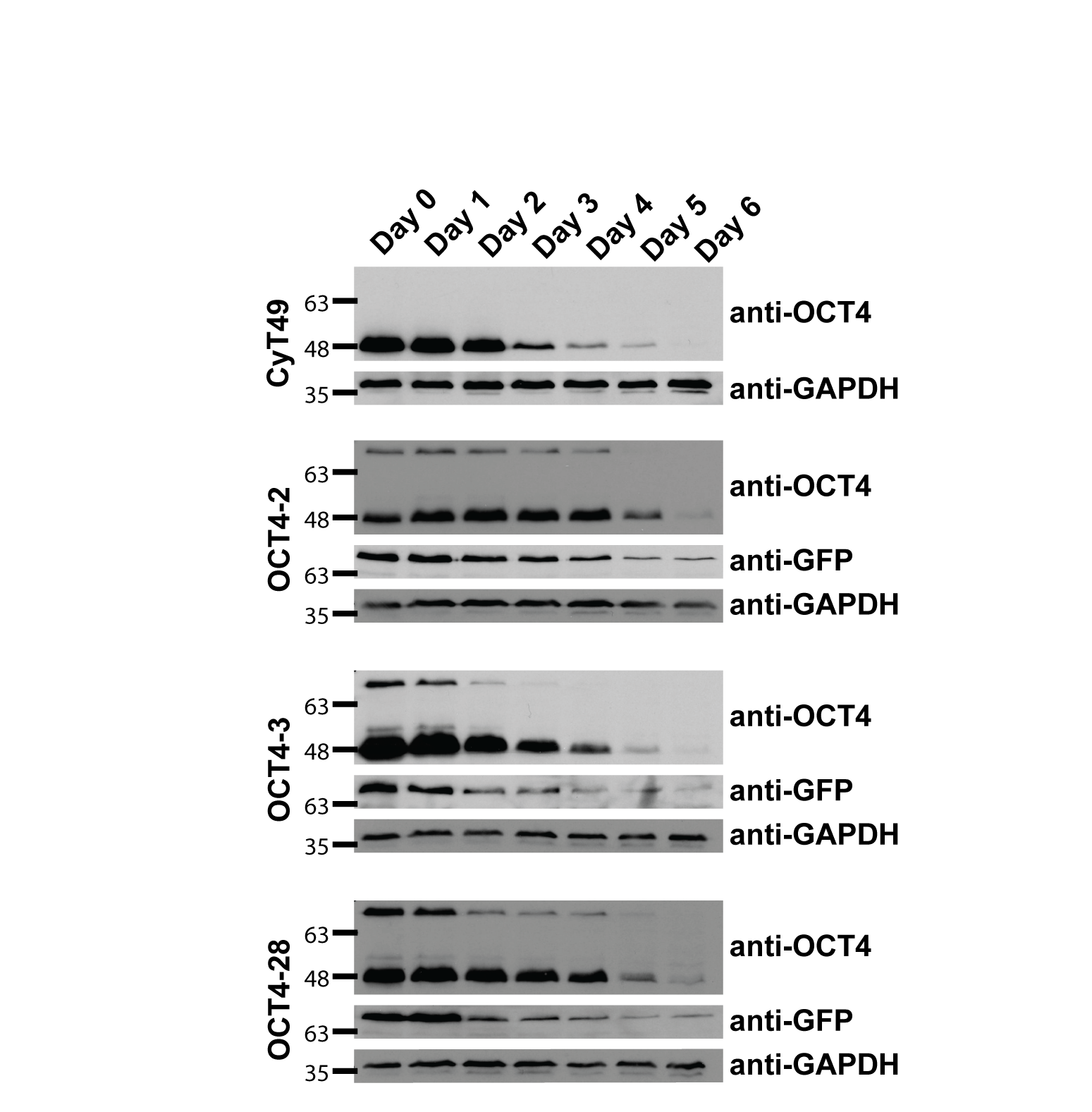

Supplement: Figure S3 — Western Blot analysis of OCT4 and eGFP expression during differentiation of hESCs to definitive endoderm and primitive gut tube. Protein lysates were collected on Day 0 (hESC), Days 1–3 (definitive endoderm) and Days 4–6 (primitive gut tube) and the expression of OCT4, eGFP and the control protein GAPDH were analyzed in the CyT49, OCT4-2, OCT4-3 and OCT4-28 hESC lines using SDS-PAGE followed by western blotting as described in the Materials and Methods section. (TIF) [file pone.0114275.s003.tif]

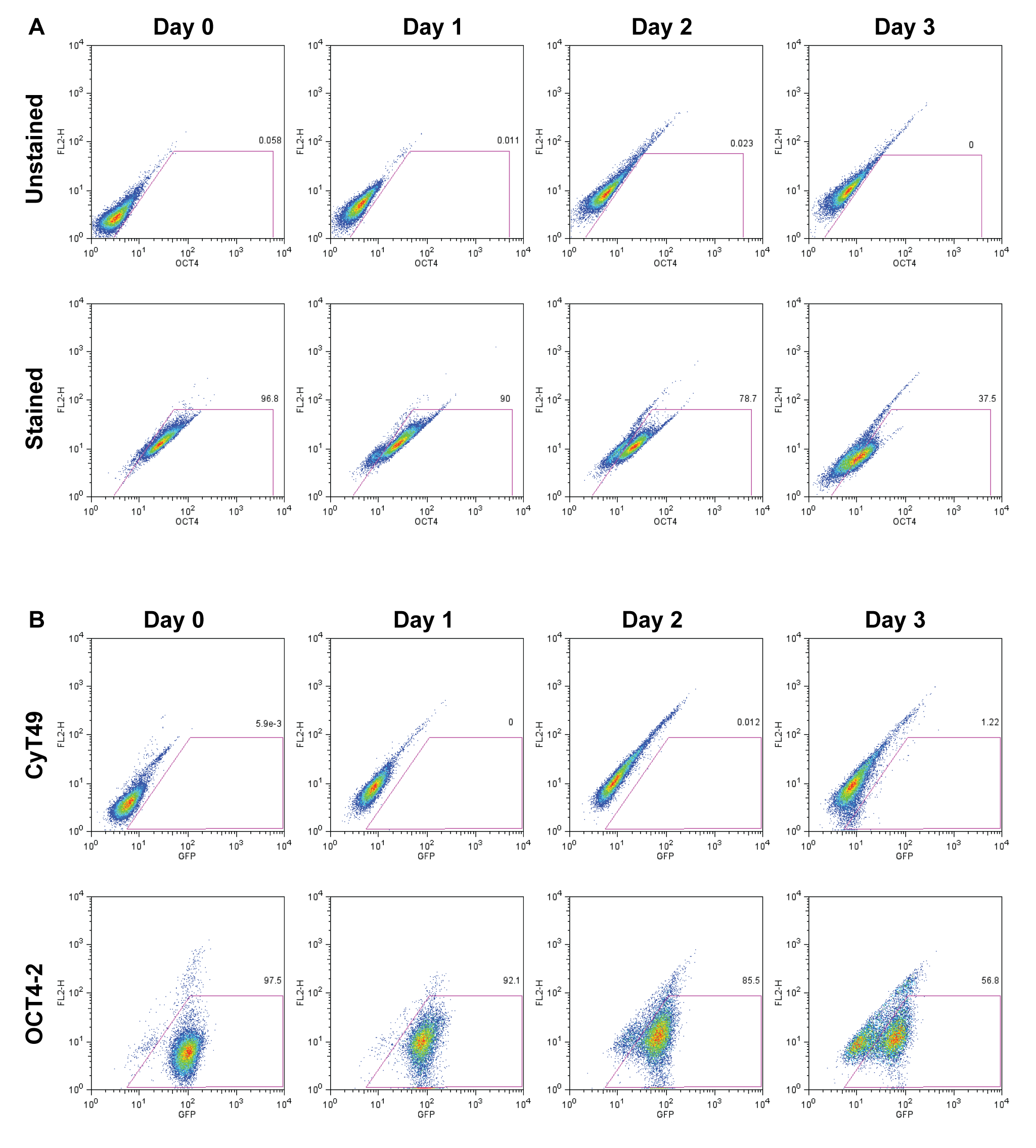

Supplement: Figure S4 — Representative flow cytometry data for the analysis of OCT4+ and eGFP+ cells. (A) Representative flow plots for CyT49 cells that were collected on Days 0–3, stained for OCT4, and analyzed using BD FACSCalibur. Unstained controls used to set up the gating strategy are also shown. (B) Representative flow plots for OCT4-2 cells that were analyzed for eGFP expression using the BD FACSCalibur on Days 0–3. To set up the gates for eGFP, CyT49 cells were used as a negative control. Data analysis was performed using FlowJo software. (TIF) [file pone.0114275.s004.tif]
