# Supplementary material for: TALEN/CRISPR-Mediated eGFP Knock-In Add-On at the OCT4 Locus Does Not Impact Differentiation of Human Embryonic Stem Cells towards Endoderm
Source: PLoS One. 2014 Dec 4;9(12):e114275. doi: 10.1371/journal.pone.0114275 (PMC4256397; doi:10.1371/journal.pone.0114275)
Supplement: Table S1 — List of Taqman qPCR primers. (PDF) [file pone.0114275.s005.pdf]

**GENE NAME**	**PROBE**	**PRIMER 1**	**PRIMER 2**	**DYE**
CER1	TAAGCTCTTACACCCACCATCCCACT	TGCAGTTCGCCTTCACTATGGACT	GATTTGCGGGACAAAGGGCAAGAT	FAM/IABLKFQ
GSC	CAAGAAATGCTTCCGGGCTGGC	TGGACAAAGACAAGAGGAACC	ATAGCTTGACCTTCGAGTGC	FAM/IABLKFQ
OCT4	CCCCCTGTCCCCCATTCCTAGA	TCTCCCATGCATTCAAACTGAG	CCTTTGTGTTCCCAATTCCTTC	FAM/IABLKFQ
SOX17	AGGGCATCATTCTCCGTCTGGTTG	ACTCCGTCAGTTTGTGCAG	CTGGGACAGATCTTCAGGTTC	FAM/IABLKFQ
SOX7	TCCATCAGCAAGCACTGCCGACTACAAT	TCCTCCAGGTTTGCCATCTTCAGT	TCCTCCAGGTTTGCCATCTTCAGT	FAM/IABLKFQ
TBP	TCCAAAGGATGCAGAGAAAGCCATCA	GGGAGCATTGGTGAAATAGAATC	GGTTTTGGTCTGGAGTCTGAG	FAM/ZEN/IABKFQ
